# New Approach for Correcting Noncovalent Interactions in Semiempirical Quantum Mechanical Methods: The Importance of Multiple-Orientation Sampling

**DOI:** 10.1021/acs.jctc.1c00365

**Published:** 2021-08-23

**Authors:** Sergio Pérez-Tabero, Berta Fernández, Enrique M. Cabaleiro-Lago, Emilio Martínez-Núñez, Saulo A. Vázquez

**Affiliations:** Departamento de Química Física, Facultade de Química, Universidade de Santiago de Compostela, Santiago de Compostela 15782, Spain

## Abstract

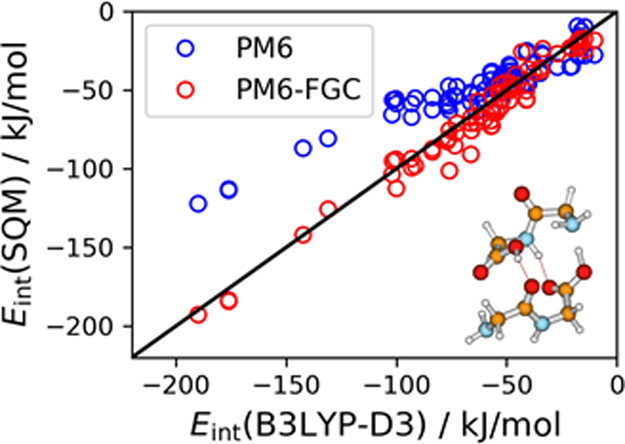

A new approach is presented to improve the performance of semiempirical quantum mechanical (SQM) methods in the description of noncovalent interactions. To show the strategy, the PM6 Hamiltonian was selected, although, in general, the procedure can be applied to other semiempirical Hamiltonians and to different methodologies. A set of small molecules were selected as representative of various functional groups, and intermolecular potential energy curves (IPECs) were evaluated for the most relevant orientations of interacting molecular pairs. Then, analytical corrections to PM6 were derived from fits to B3LYP-D3/def2-TZVP reference–PM6 interaction energy differences. IPECs provided by the B3LYP-D3/def2-TZVP combination of the electronic structure method and basis set were chosen as the reference because they are in excellent agreement with CCSD(T)/aug-cc-pVTZ curves for the studied systems. The resulting method, called PM6-FGC (from functional group corrections), significantly improves the performance of PM6 and shows the importance of including a sufficient number of orientations of the interacting molecules in the reference data set in order to obtain well-balanced descriptions.

## 1 Introduction

One of the well-known problems inherent to semiempirical quantum mechanical (SQM) methods is the poor performance in describing noncovalent interactions.^1,2^ Over the last years, much effort has been devoted to improve the accuracy of SQM methods for noncovalent interactions, particularly those based on the neglect of diatomic differential overlap (NDDO) approximation.^3,4^ The most common strategy used to ameliorate the performance of SQM methods in calculations of intermolecular interactions has been the inclusion of empirical corrections.^5−21^

Řezáč, Hobza, and their co-workers developed several generations of corrections for dispersion,^6,7,9^ hydrogen bond,^7,9^ and halogen bond^8^ interactions and parameterized them within the PM6 method^22^ as well as for other SQM methods. Contributions to this series of generations were also made by Korth^10^ and Jensen and co-workers.^11^ The final version of this series of corrections is called D3H4X, in reference to the third-generation dispersion correction, fourth-generation hydrogen-bonding correction, and halogen-bonding correction. In this version, the dispersion correction is the D3 proposed by Grimme et al. for density functional theory (DFT),^23^ but without including the 1/*r*^8^ term, which was considered to yield no significant improvement in the case of SQM methods.^9^ For these methods, Řezáč and Hobza found a specific error in the description of interactions between hydrocarbons, namely, the overestimation of interaction energies and the underestimation of equilibrium distances.^9^ To solve this problem, they included a repulsive term for all pairs of hydrogen atoms. The function used to improve the description of hydrogen bonding includes a polynomial function of degree 7 in the donor–acceptor distance, which is scaled by an angular term (dependent on the acceptor-hydrogen-donor angle) and a proton transfer term that varies with the hydrogen position. If the system contains charged groups, an additional factor is included to increase the strength of the correction. Finally, the correction used for halogen bonding consists of an exponential term.^8^ The D3H4X correction and other generations of corrections have been implemented in the MOPAC2016 program.^24^

The procedure adopted by Řezáč and Hobza to parameterize the D3H4 corrections was as follows:^9^ First, they fitted the hydrogen-bonding correction, including the contribution from dispersion in the calculated energies of the considered hydrogen-bonded complexes. For the fittings, they performed least-squares optimizations, minimizing the root-mean-square error of the interaction energy when compared to reference data obtained at the coupled cluster singles and doubles with perturbative triples correction/complete basis set (CCSD(T)/CBS) level of calculation. Specifically, as a benchmark set, they used the S66 database,^25−27^ which includes dissociation curves for 66 noncovalent complexes that exhibit dispersion, hydrogen bonds, and mixed dispersion/electrostatic interactions.

Truhlar, Gao, and their co-workers developed the polarized molecular orbital (PMO) method^12−16^ based on a NDDO Hamiltonian that includes polarization functions on hydrogen atoms. In addition, to improve the description of dispersion interactions, they added the first damped dispersion term developed by Grimme.^28,29^ This dispersion correction was previously used by Hillier and co-workers^5,20^ in conjunction with the AM1^30^ and the PM3^31^ Hamiltonians. The final versions of the PMO method, that is, PMO2^15^ and PMO2a,^16^ have been found to accurately describe polarization effects as well as noncovalent complexation energies. The PMO2 method was parametrized for all compounds containing H, C, and O atoms, and the PMO2a version is an extension of PMO2 to new functionalities, which includes parameters for amino nitrogen groups and molecules containing sulfur–oxygen bonds. Parameterizations of the PMO Hamiltonians were carried out using a genetic algorithm, which has the advantage of efficiently exploring the search space to find near optimal solutions when the number of fitting parameters is large. The abovementioned PMO versions have been implemented on the MOPAC 5.022mn package.^32^

The work of Thiel and co-workers directed to improve the reliability of SQM methods also deserves some attention. They developed the orthogonalization-corrected methods OMx^33−35^ and ODMx,^17^ which include significant improvements in the semiempirical Hamiltonian, thus leading, in general, to better results in comparison with NDDO-based methods that make use of the modified neglect of diatomic overlap (e.g., AM1 or PM6). These semiempirical Hamiltonians needed to incorporate dispersion corrections to improve the description of noncovalent interactions. In particular, they include Grimme’s D3 dispersion correction^23,36^ with the Becke–Johnson damping function,^37−39^ as well as Axilrod–Teller–Muto three-body terms,^23,40^ which ameliorate the description of large dense systems.^41,42^ For the recent ODMx methods, several training sets were used, including the abovementioned S66 data set.^25−27^ Parameterization of semiempirical Hamiltonians and correction potentials for noncovalent interactions is a key issue, and the procedure followed within the ODMx methods is extensively discussed in the recent article by Dral et al.^17^

The abovementioned studies led to remarkable improvements in SQM methods for the evaluation of noncovalent interactions. In general, the corrections for dispersion and hydrogen bonding interactions are modeled by potential functions based on physically sound formulas. In addition, the training sets used for parameterizations are quite large, which may ensure a wide range of applicability. However, and as it will be shown later in the present work, the errors in the description of noncovalent interactions may be significant, depending on the relative orientation of the interacting molecules. This can be a consequence of possible shortcomings in popular data sets, which in general only include the most relevant configurations of interacting molecules.

In this paper, we present, as a proof-of-concept study, an alternative way to develop analytical corrections for SQM methods to improve the description of noncovalent interactions. The idea is based on previous chemical dynamics studies in which pairwise intermolecular potentials were parameterized through fittings to a series of intermolecular potential energy curves (IPECs) that emphasize the different atom-pair potentials exhibited by the interacting molecules.^43−47^ Following the strategy used to develop potentials for interactions of peptides with self-assembled monolayers of perfluorinated alkanes,^47^ we selected small molecules as representatives for typical functional groups and evaluated IPECs for all possible molecular pairings. Specifically, for this proof-of-concept study, we have chosen methane, formic acid, and ammonia, which give six different pair combinations: the three dimers and the CH_4_/HCO_2_H, CH_4_/NH_3_, and NH_3_/HCO_2_H pairs of molecules. We developed empirical corrections for the PM6 method, which in principle can be used for interactions between hydrocarbons, carboxylic acids, and amines. To assess the performance of the method as well as the transferability of the corrections to other systems, we applied them to evaluate interaction energies for several complexes of the S66,^25^ A24,^48^ and ADIM6^49,50^ data sets, as well as for a collection of different conformers of the diglycine dimer and trimer, and the dialanine dimer, obtained through automated exploration of the corresponding potential energy surfaces (PESs). The novelty of our approach is the introduction of two important features. First, and most important, the inclusion of several orientations of the interacting molecules in the database, which is crucial to obtain well-balanced corrections. Second, the use of general corrections to take into account that SQM methods have significant limitations to accurately describe not only dispersion interactions but also electrostatics, induction, and exchange repulsion. We notice that the purpose of this paper is not to present corrections with final parameters for universal applicability, but to show a strategy to develop corrections that can satisfactorily model noncovalent interactions for all orientations of interacting molecules.

## 2 Methods

Intermolecular potential energy curves for the six pairs of molecules indicated above were calculated using CCSD(T)^51^ and the augmented correlation consistent polarized valence triple-zeta basis set aug-cc-pVTZ.^52^ The IPECs were also evaluated employing DFT with the B3LYP functional,^53−55^ including the D3 dispersion correction with the Becke–Johnson damping scheme^37−39^ and with the valence triple-zeta polarization def2-TZVP basis set.^56^ The IPECs were computed using the supermolecular approach with frozen intramolecular geometries and correcting the interaction energy for basis set superposition error (BSSE) through the counterpoise method.^57,58^ The intramolecular geometries were obtained by B3LYP-D3/def2-TZVP optimizations. Several orientations of the interacting molecules were selected to stress the different pair-type interactions. Specifically, for each pair of molecules, the number of orientations was at least equal to the number of the different pair-type interactions. A proper selection of orientations is crucial to obtain well-balanced corrections. These electronic structure calculations were performed with the ORCA 4.0 program and the default frozen core approximation.^59,60^

The general expression of the noncovalent potential–energy correction developed in this work for the PM6 method is written as a pairwise sum of the form
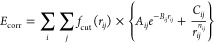
1where indexes *i* and *j* refer to atoms belonging to different interacting molecules, and *r*_*ij*_ is the interatomic distance between atoms *i* and *j*. Parameters *A*_*ij*_, *B*_*ij*_, and *C*_*ij*_ (real numbers) as well as *n*_*ij*_ (integers) depend on the nature of the considered pair of atoms. *f*_cut_(*r*_*ij*_) is a cutoff function introduced to remove the correction at very short *r*_*ij*_ distances
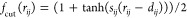
2where *s*_*ij*_ is a parameter that controls the strength of the damping for the interaction between atoms *i* and *j*, and *d*_*ij*_ is the distance at which the cutoff function takes the value 1/2. The *n*_*ij*_ parameters were not fixed to 6; rather, they were allowed to vary around this value. Also, the *A*_*ij*_ and *C*_*ij*_ parameters may be either positive or negative. We notice that eq 1 should be regarded as a practical correction, without any physical interpretation. However, one may expect the functional form given by eq 1, based on Buckingham’s potential,^61^ to work reasonably well because this potential can model intermolecular interactions with pretty good accuracy.

The abovementioned parameters were obtained through fittings to differences between the interaction energies calculated at the reference level and those computed with the PM6 method. As described in the next section, the IPECs obtained by B3LYP-D3/def2-TZVP calculations are in very good agreement with those determined at the CCSD(T)/aug-cc-pVTZ level of theory, showing the efficiency of the combination of the B3LYP-D3 density functional and the def2-TZVP basis set in providing acceptable and inexpensive IPECs. For our approach, the use of an accurate and inexpensive reference methodology is important because, in general, we need a thorough exploration of potential energy surfaces for both parameterization and validation processes. For this reason and considering that future work, for extending the method to other types of functional groups, will involve a large amount of calculations, we have selected the B3LYP-D3/def2-TZVP level as the reference for the fittings. Furthermore, for the molecular systems considered in this study, the errors of the fits are larger than the B3LYP-D3–CCSD(T) potential energy differences. The SQM calculations were carried out with the MOPAC2016 program.^24^ We used a least-squares nonlinear fitting procedure based on a genetic algorithm, as implemented in our GAFit code,^62^ and with the following objective function, χ^2^
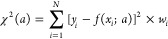
3where (*x*_*i*_, *y*_*i*_) represents one of the *N* data points, a is the collective variable formed by the total number of fitting parameters, and *f* (*x*_*i*_; a) is the value of the model function at *x*_*i*_ (i.e., a particular geometry of the interacting molecules). The square of the difference between *y*_*i*_ (i.e., a B3LYP-D3–PM6 energy difference) and the corresponding model value, calculated with eqs 1 and 2, may be multiplied by a weighting factor (*w*_*i*_) assigned to each data point.

To validate our model function and parameterization strategy, as well as to explore the transferability of the corrections, we applied them to several complexes of the S66,^25^ A24,^48^ and ADIM6^49,50^ databases. Moreover, we checked the performance of the corrections on a data set formed by a collection of different conformations of diglycine and dialanine complexes, obtained by an automated exploration of the PESs at the PM6-D3H4 level, using the AutoMeKin package,^63−65^ which has an interface with the MOPAC2016 program.^24^ Specifically, we considered the diglycine dimer and trimer, as well as the dialanine dimer. Although AutoMeKin has mainly been designed to discover and simulate chemical reaction mechanisms, it includes an option for obtaining stationary points for intermolecular complexes.^66^ As for the fittings, the benchmark level for this validation was B3LYP-D3/def2-TZVP, correcting the interaction energies for BSSE.

## 3 Results and Discussion

### 3.1 Formic Acid Dimer

**Figure 1:**
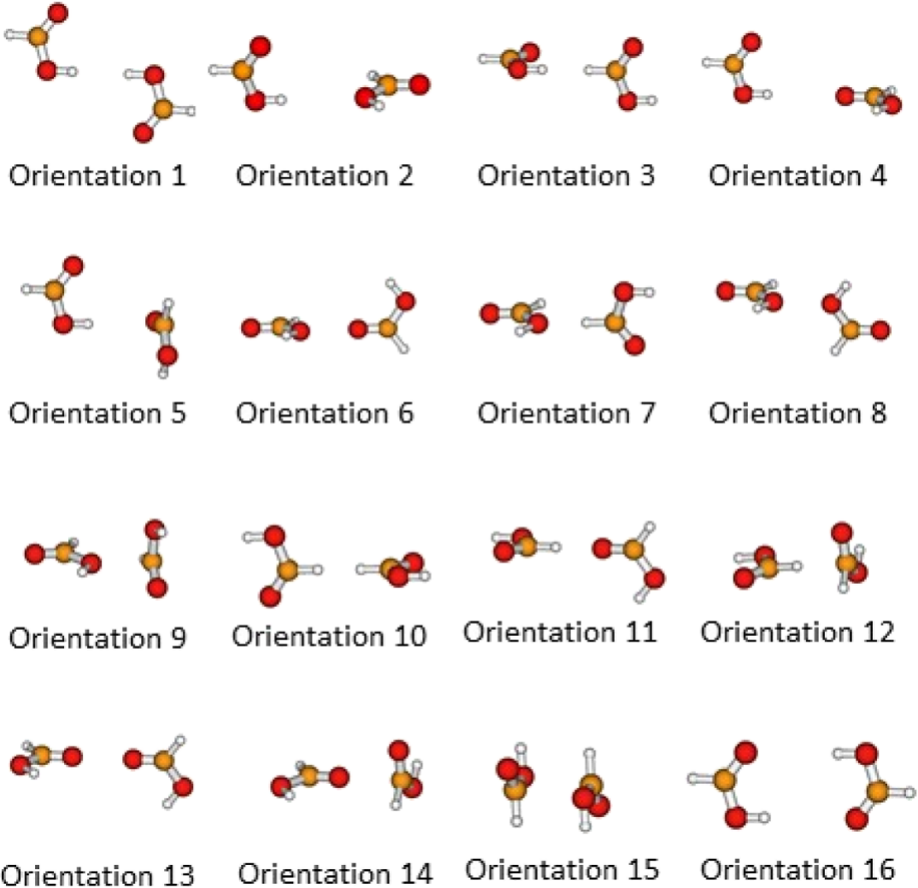
Orientations of the formic acid dimer considered for the fitting.

The formic acid molecule has five chemically nonequivalent atoms, that is, all the atoms are nonequivalent. Therefore, for this system, there are 15 different types of pairwise interactions. Consequently, for the fittings, we included 15 orientations that emphasize the distinct pairwise interactions, as well as an additional orientation corresponding to the global minimum of the formic acid dimer, which exhibits double hydrogen bonding. These 16 orientations are depicted in Figure 1.

**Figure 2:**
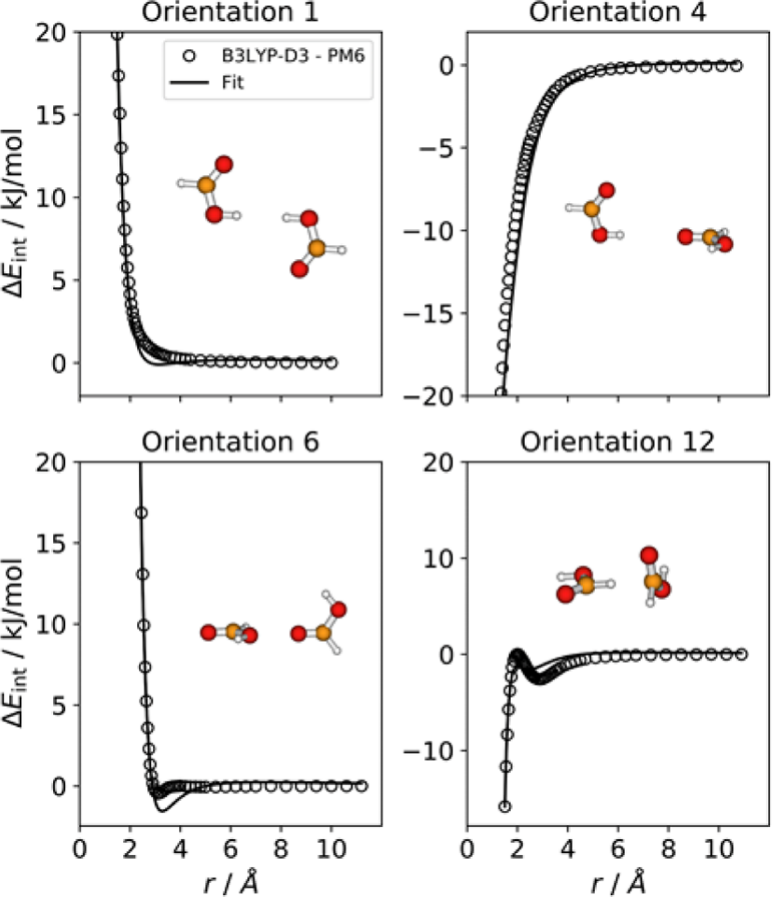
B3LYP-D3–PM6 interaction energy differences (open circles) for selected orientations of the formic acid dimer. The black lines correspond to the fit (see text).

**Figure 3:**
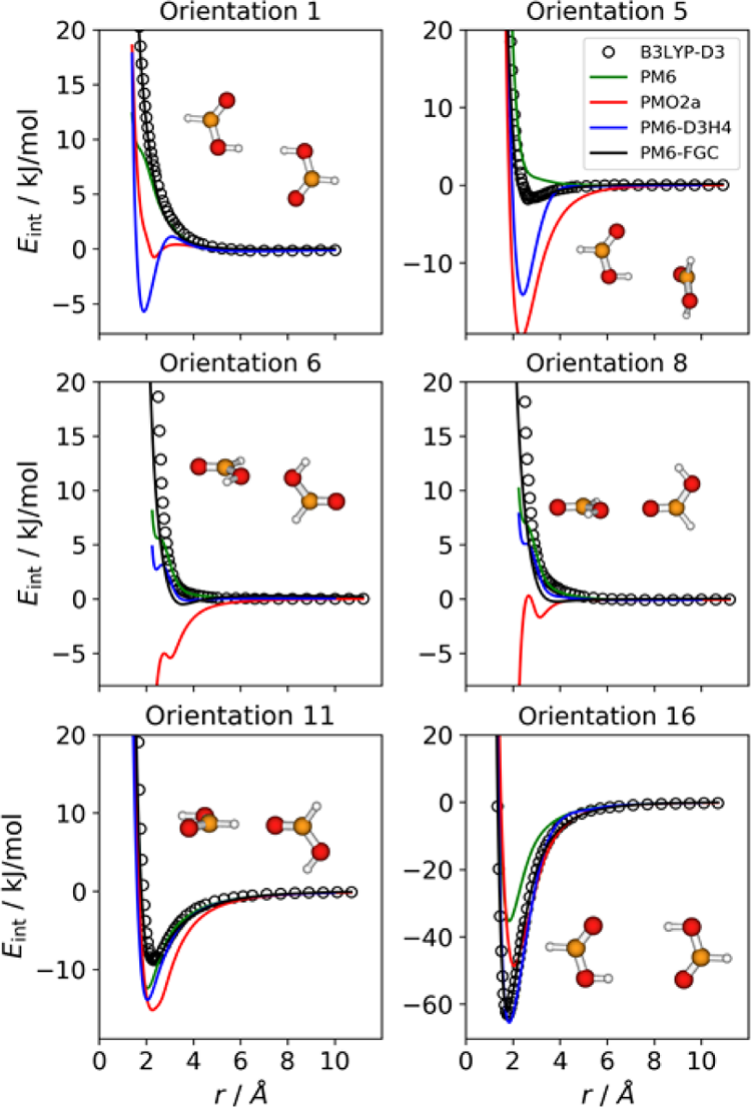
Comparison of IPECs for six selected orientations of the formic acid dimer.

As pointed out in the previous section, for the selected orientations, we calculated intermolecular potential energy curves at the CCSD(T)/aug-cc-pVTZ and B3LYP-D3/def2-TZVP levels. As shown in the Supporting Information, Figures S1–S7, the agreement between the CCSD(T) and DFT curves is excellent. To develop corrections for the PM6 method, using the functional form specified in eqs 1 and 2, we used the differences between the B3LYP-D3/def2-TZVP interaction energies and the corresponding PM6 values as the data for the fittings. These energy differences have the typical forms displayed in Figure 2. In these four plots, *r* corresponds to the distance between attacking atoms, that is, the two carboxylic hydrogens in orientation 1, for example. The form of the energy difference as a function of *r* for this orientation resembles a typical repulsive potential, indicating that PM6 has a less repulsive IPEC than that of the reference method. By contrast, for orientation 4, the form of the DFT–PM6 plot behaves as a decaying exponential with negative amplitude (*A*_*ij*_ in eq 1), thus pointing out a stronger repulsion character of the PM6 interaction potential. Most of the orientations show a well followed by a pronounced increase of the energy difference as the distance between the attacking atoms becomes shorter, as can be seen for orientation 6 (carbonyl oxygen–hydroxyl oxygen attack). The presence of a well in these plots does not mean that the DFT and PM6 IPECs exhibit potential minima (although in most cases they do). Actually, for orientation 6, the DFT and PM6 IPECs are repulsive in nature, as can be seen in Figure 3. In very few cases (only one for the formic acid dimer), the B3LYP-D3–PM6 energy differences display a more complex form, showing both a minimum and a maximum, as for orientation 12, where the hydrogen attached to the carbonyl carbon attacks the carbon atom of the other molecule. Although it could be expected that eq 1 would be valid as a practical and simple correction for SQM methods, these plots further justify its use.

**Figure 4:**
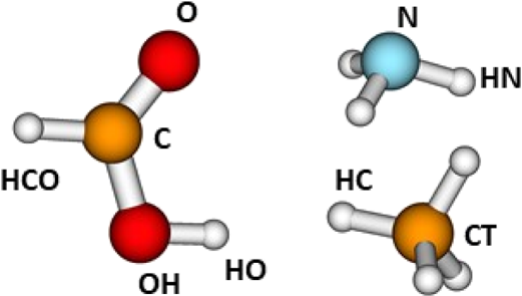
Atom types defined for ammonia, formic acid, and methane.

Using GAFit^62^ and the geometries and energy differences corresponding to the 16 orientations of the formic acid dimer, we simultaneously fitted the parameters involved in eqs 1 and 2. There is not a universal, objective way to conduct a parametrization and, furthermore, with the use of genetic algorithms, one may obtain many solutions than can be equally valid. For general discussions on parameterizations for SQM methods, the reader may consult the studies on the development of the PMO2a^16^ and ODMx^17^ methods. For the formic acid dimer, because there are 15 different types of pairwise interactions, the total number of parameters is 60, without including those associated with the cutoff function given by eq 2. All the 60 parameters were fitted simultaneously. We found that including the cutoff parameters into the parameter spectrum explored by the genetic algorithm did not improve the fittings significantly. For this reason, after some analyses and to avoid overparameterization, we have chosen a value of 10 for all the *s*_*ij*_ parameters and different values for the *d*_*ij*_ parameters, depending on the nature of atoms *i* and *j*. The parameters obtained from our best fit are collected in Table 1, and the fit results are depicted in Figure 2 for some selected orientations. For simplicity, for parameters *A*, *B* and *C*, we only show two decimals; the high precision parameters are included in Table S1 in the Supporting Information. Notice that we have defined atom types in much the same way as in molecular mechanics force fields. The symbols chosen in this work are shown in Figure 4. Adding to the PM6 interaction potential the corrections calculated with eqs 1 and 2, and the parameters fitted in this work, results in our PM6-FGC method. We have chosen the FGC acronym, from functional group corrections, to emphasize the idea of specific parameters being obtained for different functional groups.

**Table 1:** Parameters Obtained for the Formic Acid Dimer^a^

atom pair	*A*	*B*	*C*	*n*	*d*
C–C	87222.68	4.52	–2614.24	6	1.8
C–O	18423.16	3.22	–4.76	10	1.7
C–OH	101988.82	3.76	–801.90	6	1.7
C–HO	–118682.03	5.31	49.92	4	1.2
C–HCO	–91515.91	5.31	5.68	2	1.2
O–O	157533.31	3.84	–249.70	9	1.7
O–OH	229576.11	3.74	–1494.51	6	1.7
O–HO	–12321.28	5.15	–106.36	3	1.0
O–HCO	7911.47	3.45	–735.00	9	1.2
OH–OH	282168.52	3.74	–1073.49	5	1.7
OH–HO	–3746.89	3.44	–49.80	4	1.0
OH–HCO	3900.44	2.91	–380.87	8	1.2
HO–HO	2029.79	3.07	27.04	2	1.0
HO–HCO	10653.65	4.26	–396.77	11	1.0
HCO–HCO	9870.51	3.49	–289.72	5	1.0

a The units are such that the potential energy is in kJ/mol and distances in Å.

Figure 3 compares the reference IPECs with the PM6-FGC interaction curves for six selected orientations of the formic acid dimer. The global minimum corresponds to orientation 16. The IPECs for the remaining orientations are displayed in Figure S8 in the Supporting Information. For comparison, we include the PM6 curves as well as those obtained with the PM6-D3H4^9^ and PMO2a^16^ methods, which are implemented in the freely distributed MOPAC2016^24^ and MOPAC 5.022mn^32^ programs, respectively. It would be interesting to include results of calculations using the ODMx method;^17^ however, to our knowledge, the code in which this method is implemented is not freely available.^67^ As can be seen from Figures 3 and S8, the PM6-FGC curves (black lines) agree well with the B3LYP-D3/def2-TZVP data (black open circles).

The IPECs calculated with the PMO2a method (red lines) display remarkable discrepancies with the reference curves. Strikingly, for orientations 6 and 8, as well as for orientation 13 (see Supporting Information), this method shows an unphysical behavior, because the interaction energy in the repulsive region decreases as the distance between the attacking atoms decreases. These orientations correspond to configurations that emphasize the interaction between oxygen atoms. Clearly, a revision of this method is required to improve its performance. For this reason, for the remaining systems under investigation here, we have not considered the PMO2a method any further.

The PM6-D3H4 potential energy curves are displayed as blue lines in the figures. The D3H4 corrections were developed using a training set based on CCSD(T)/CBS data, so that slight deviations may be expected because our IPECs were obtained with B3LYP-D3/def2-TZVP calculations, although, as already mentioned, they are in excellent agreement with the corresponding CCSD(T)/aug-cc-pVTZ curves. As can be seen, for orientation 1, which corresponds to the attack between carboxyl hydrogens, the PM6-D3H4 method exhibits a clear minimum. This orientation is predicted to be repulsive with both B3LYP-D3 and CCSD(T) methods. Also, for orientation 5, the PM6-D3H4 method gives a significant minimum, which contrasts with the small well depth predicted by the reference calculations. It is also worth mentioning that, for orientations 6 and 8, PM6-D3H4 and PM6 exhibit an unphysical behavior in the repulsive region (around 5 kJ/mol). Although the S66x8 data set^25^ employed by Hobza and co-workers comprises a wide range of complexes, and the D3H4 corrections are able to describe noncovalent interactions for the most relevant orientations of interacting molecules, our results point out some deficiencies in these corrections, which may be especially problematic for dynamics studies, where all orientations may be sampled. The source of these deficiencies comes from an important drawback of the S66x8 database, namely, the fact that, in general, it only includes the most relevant orientation for each selected complex. For carboxylic acids, this database includes the acetic acid dimer in its most attractive orientation, that is, that exhibiting a double hydrogen bond (the equivalent of orientation 16 for the formic acid dimer, Figure 1).

### 3.2 Ammonia Dimer

**Figure 5:**
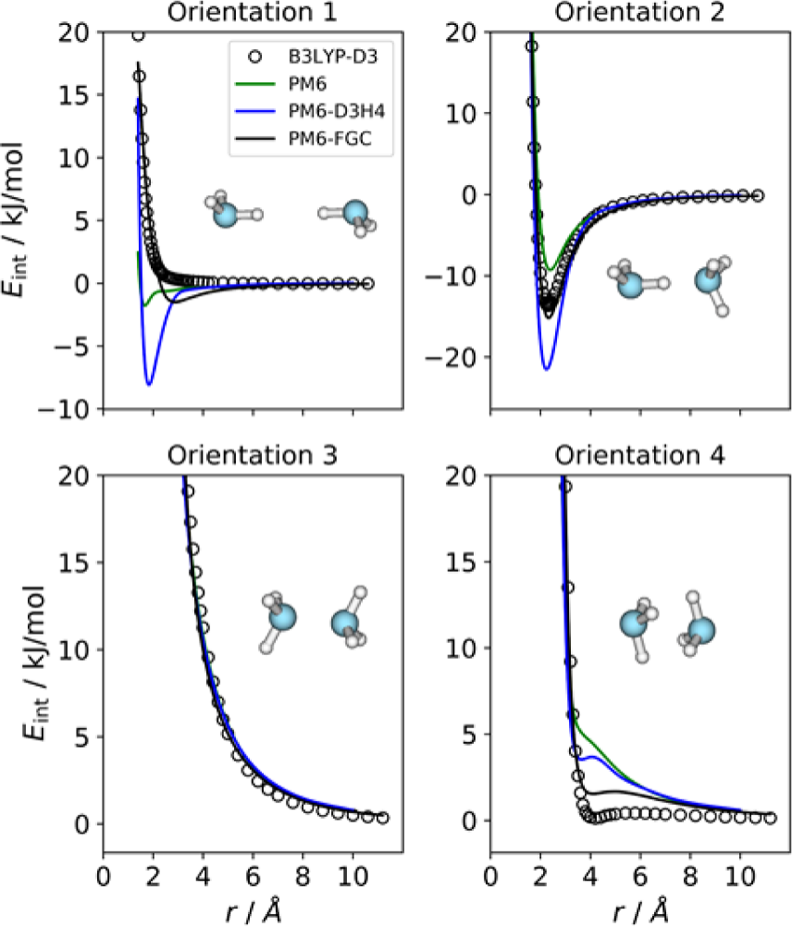
Comparison of IPECs for the considered orientations of the ammonia dimer.

This dimer shows three different types of pairwise interactions, and therefore, we need at least three different orientations. In this work, we considered the four orientations depicted in Figure 5, which compare the IPECs obtained with PM6 (green lines), PM6-D3H4 (blue lines), and PM6-FGC (black lines), with those determined with the reference method (open circles). As can be seen from Figure 5, the PM6-D3H4 method exhibits substantial deficiencies, similar to those encountered in the formic acid dimer. Specifically, it predicts a remarkable minimum for orientation 1 (H···H attack), which is clearly repulsive at the reference level. In addition, for orientation 2, which exhibits hydrogen bonding, the PM6-D3H4 method clearly overestimates the strength of the interaction.

Among the systems considered in this study, the ammonia dimer was the most challenging. Actually, using our simple expression for the analytical correction (eqs 1 and 2), we were not successful at obtaining a good fit, as reflected in Figure 5. Particularly, for orientation 4, PM6-FGC as well as PM6 and PM6-D3H4 show curves more repulsive than that predicted with the benchmark method. One way to improve the fit is to add a pseudoatom to model the effect of the nitrogen lone pair, as it is done in several force fields, but for this proof-of-concept presentation, we wanted to keep the correction scheme as simple as possible. The parameters obtained from the ammonia dimer fit are collected in Table S1.

### 3.3 Methane Dimer

**Figure 6:**
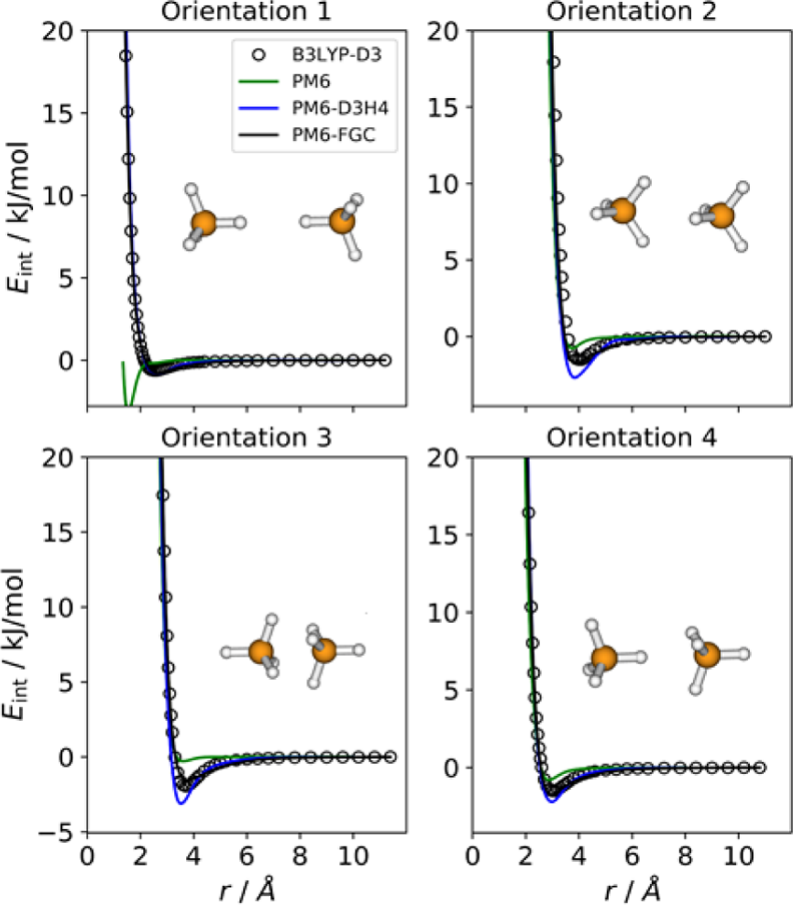
Comparison of IPECs for the considered orientations of the methane dimer.

The IPECs evaluated for this dimer are displayed in Figure 6, which also describes the orientations selected in this work. In principle, this system appears to be the simplest one among those studied here. As can be seen, both PM6-D3H4 and PM6-FGC, using the parameters shown in Table S1, exhibit IPECs in satisfactory agreement with the reference curves. The underestimation of the dispersion interaction in the PM6 method is clear, but the worse performance predicted with this method appears for orientation 1 (i.e., H···H attack), which shows a significant minimum at a quite short H···H distance.

### 3.4 Ammonia–Formic Acid Complex

**Figure 7:**
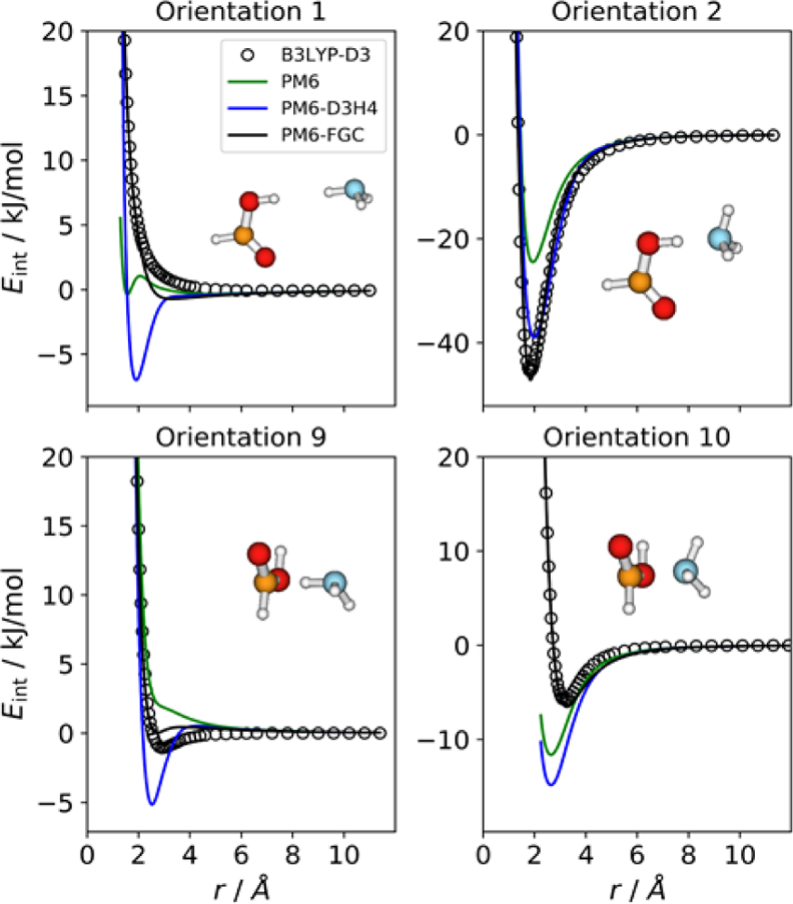
Comparison of IPECs for four selected orientations of the HCOOH/NH_3_ complex.

For this complex, we considered 10 orientations, that is, the same number as that of the different types of pairwise interactions. The best fit for this system led to the parameters reported in Table S1. Figure 7 depicts four selected orientations, together with their IPECs. The plots for the remaining orientations are shown in Figure S9. The most attractive orientation exhibits a hydrogen bond between the carboxylic hydrogen and the ammonia nitrogen (orientation 2). Both the PM6-D3H4 (blue line) and the PM6-FGC (black line) methods satisfactorily describe the interaction for this orientation. However, for several other orientations (1, 5, 7, 9, and 10), the PM6-D3H4 method predicts minima with potential well depths larger than those obtained through CCSD(T) and B3LYP-D3 calculations.

For orientation 1, that is, the attack between carboxylic and ammonia hydrogens, the behavior of the PM6-D3H4 curve resembles that found for orientation 1 in the formic acid and ammonia dimers. In these three cases, the reference IPECs clearly exhibit a repulsive character. The PM6-FGC curve shows a small deviation from the reference IPEC, similar to that exhibited in the ammonia dimer. For orientations 9 and 10, which correspond to the attack of the carbonyl carbon to ammonia hydrogen and nitrogen, respectively, the PM6-D3H4 (and PM6) curves also show remarkable discrepancies with respect to the reference curves. For orientation 9, the PM6-FGC curve exhibits a small deviation from the benchmark IPEC. Overall (see also Figure S9), the PM6-FGC method gives a satisfactory description of the noncovalent interaction in the ammonia-formic acid complex.

### 3.5 Methane–Formic Acid Complex

**Figure 8:**
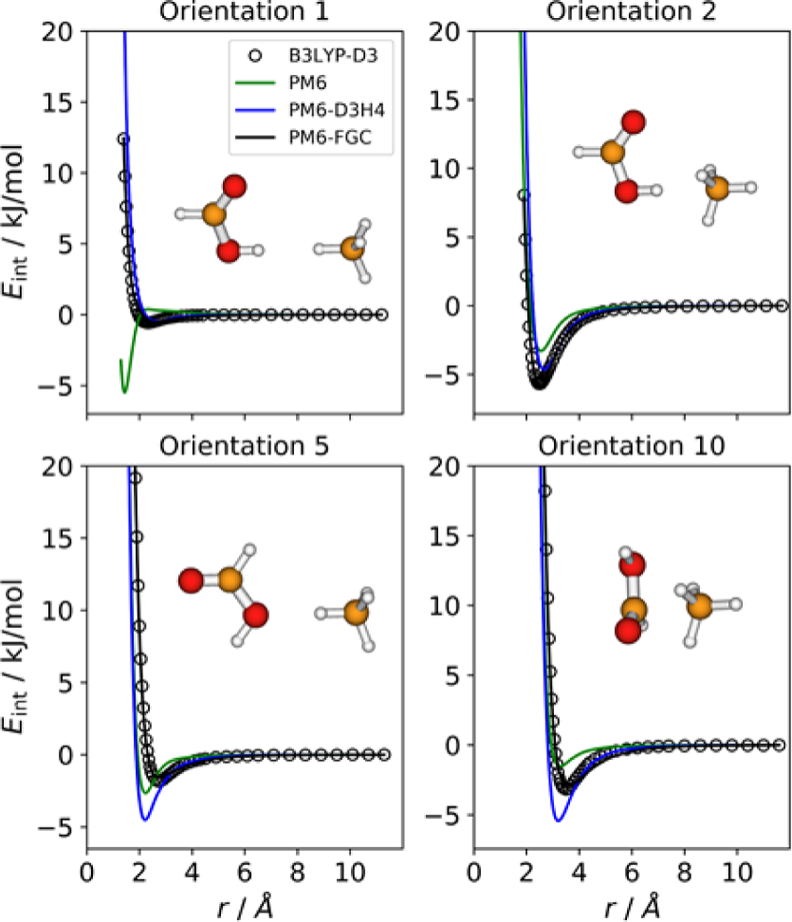
Comparison of IPECs for four selected orientations of the HCOOH/CH_4_ complex.

For this complex, we considered 10 orientations, and four of them are depicted in Figure 8, together with their corresponding IPECs. The IPECs of the remaining orientations are shown in Figure S3, and the parameters obtained in the fit are displayed in Table S1. As can be seen, the PM6-FGC curves agree well with the corresponding benchmark IPECs. The PM6-D3H4 method shows, in general, satisfactory performance, although for several orientations (e.g., 5 and 10), it predicts more attractive interactions. As expected, the PM6 interaction energies are very inaccurate, and for orientations 1 and 3 (attacks of hydrogen atoms), the corresponding IPECs display remarkable minima.

### 3.6 Ammonia–Methane Complex

**Figure 9:**
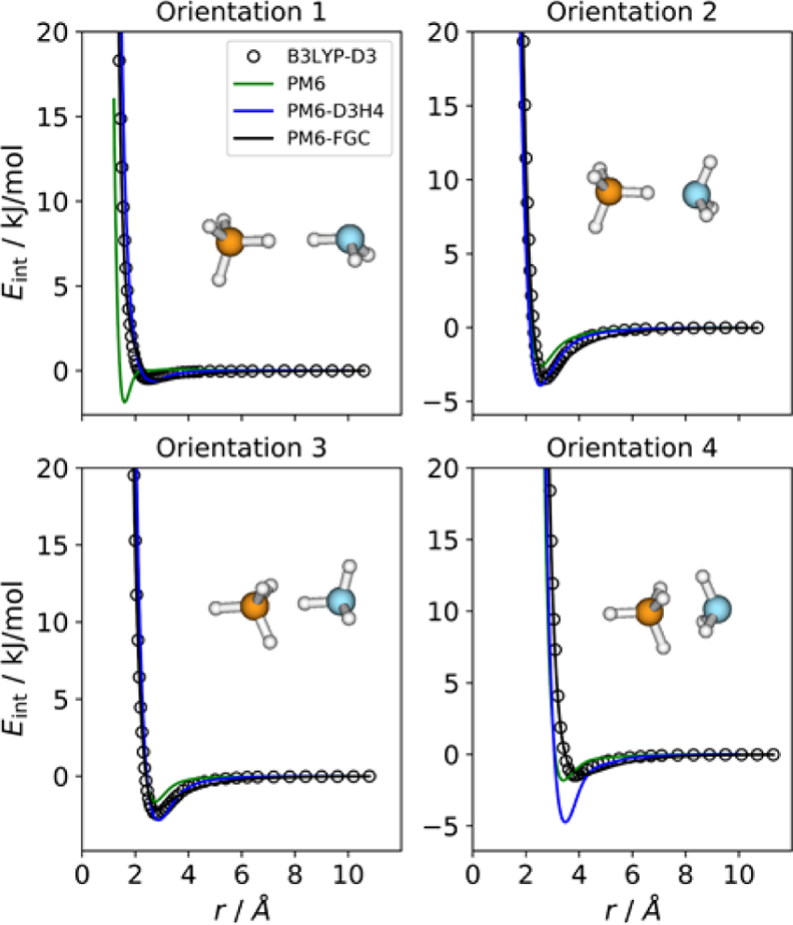
Comparison of IPECs for the considered orientations of the CH_4_/NH_3_ complex.

Four different orientations were considered for this complex, and they are displayed in Figure 9, together with their IPECs. The agreement between the IPECs obtained with the PM6-FGC method and those evaluated at the reference level reflects the good quality of the fit. The PM6-D3H4 method also predicts satisfactory interaction energies, except for orientation 4, for which it provides a significant potential well depth.

### 3.7 Validation and Critical Assessment of the FGC Approach

There are two critical issues that we need to consider in order to validate our method: (1) the possibility of overfitting and (2) parameter transferability. Overfitting occurs when the model function uses more parameters than are necessary or exhibits a more complex form than is needed.^68^ Considering the drastic approximations inherent to SQM methods, it is clear that these approaches fail in describing not only dispersion interactions but also electrostatic, induction, and exchange-repulsion. Our analytical correction consists of a pairwise sum over interacting atoms, with four parameters per interaction type. The form of the energy differences between the interaction energies calculated at the reference level and those obtained with the PM6 Hamiltonian, as exemplified in Figure 2 for selected orientations of the formic acid dimer, suggests that our approach is reasonable. Therefore, the remaining question is whether our model function and parameter dimensionality are appropriate to correct the PM6 deficiencies, without leading to overfitting. At this point, we want to remark the importance of including many orientations in the training set. Using a single orientation seems to be insufficient to parameterize well-balanced corrections.

To analyze the possibility of overfitting in our approach, let us focus on the formic acid dimer. For the fittings, we have considered that all the atoms are different, which led to 60 parameters in all, and they were simultaneously fitted using IPECs for 16 different orientations. The additional 15 parameters of the cutoff function were set, rather than fitted, after some exploratory fittings. To investigate overfitting, we have performed two sets of additional fittings: (i) considering that all oxygen atoms share the same parameters (4 atom types) and (ii) considering that, in addition, the hydrogen atoms are equivalent to each other (3 atom types). These simplifications lead to 40 and 24 parameters, respectively. For the fittings, we used the same number of points and weights as for the fittings described previously for this system (in particular, each data point had a weight of unity). The data points extended up to repulsive energies ranging from 23 to 141 kJ/mol, depending on the orientation. With the corrections obtained from the best fits (parameters shown in Tables S2 and S3), we evaluated the IPECs for the 16 orientations of the formic acid dimer, and they are plotted in Figures S11 and S12, together with the B3LYP-D3 curves and those determined with the parameters of Table 1 (5 atom types). As can be seen, when we use 4 atom types, the calculated IPECs are very similar to those evaluated with the parameters of Table 1 (i.e., five atom types). This indicates that the two oxygen atoms are quite similar two each other, as far as interaction potentials are concerned. However, when we use only 3 atom types, the corrections added to the PM6 Hamiltonian cannot accurately describe the noncovalent interactions in this dimer because significant deviations from the reference curves are found for several orientations. Therefore, the HO and HCO atom types differ significantly from each other. To quantify the deviations from the B3LYP-D3 curves, we calculated the mean absolute errors (MAEs), for the three PM6-corrected IPECs, obtaining 0.7, 0.9, and 2.8 kJ/mol for the curves determined using 5, 4, and 3 atom types, respectively.

In addition to the previous analysis, we evaluated IPECs for 10 random orientations of the formic acid dimer, which were not included in the fittings. The comparison of these IPECs is shown graphically in Figure S13. As can be seen, the PM6-FGC curves (using the parameters given in Table S1) are, in general, in good agreement with the DFT curves. For orientation 4, the PM6-FGC curve displays a potential well less pronounced than that of the reference level. The most important deviations are exhibited by the PM6-D3H4 curves for orientations 3 and 7. Notice that, for several orientations, both PM6 and PM6-D3H4 curves deviate significantly from the reference curves in the repulsive region. Finally, we notice that the MAE calculated for the PM6-FGC curves, including all the points with interaction energies up to 100 kJ/mol, is 1.1 kJ/mol, slightly smaller than that calculated employing the FGC parameters obtained using 4 atom types (1.3 kJ/mol), thus resembling the results reached for the training set. From the discussion in this and the previous paragraph, we conclude that our parameterization model does not exhibit overfitting, at least at a significant level.

**Figure 10:**
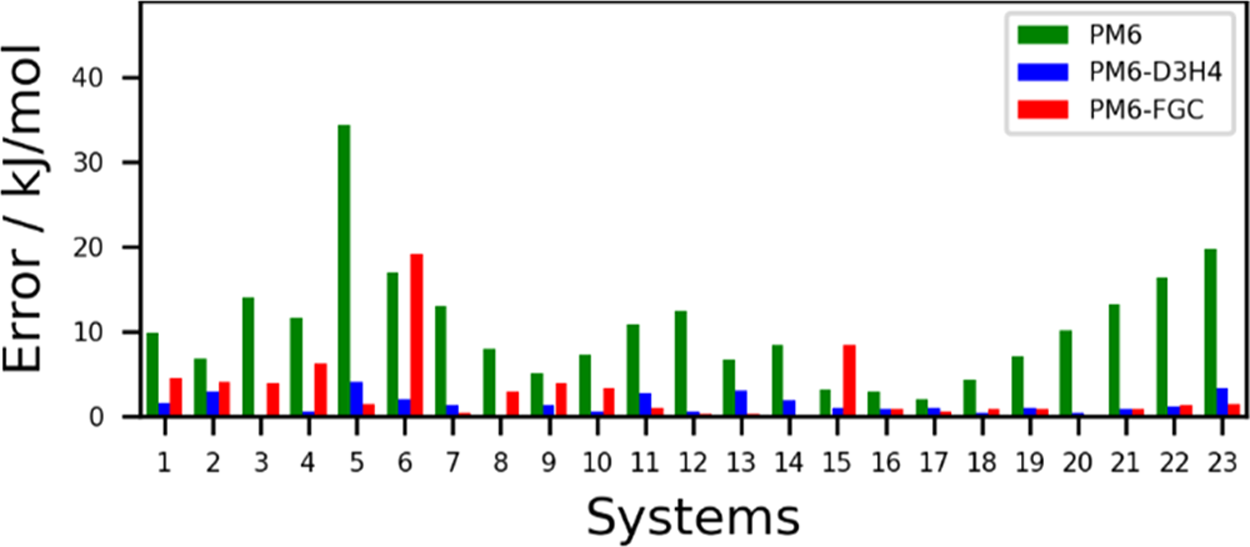
Absolute errors calculated for 23 complexes of well-known data sets. The complex associated with each system number is specified in Table S4. The benchmark interaction energies were taken from the BEGDB web page.^69^

As a further validation of our method, and particularly to analyze the transferability of the corrections to related systems, we calculated the interaction energies for 23 complexes defined in well-known databases^25^ and compared the results with benchmark data taken from the GMTKN55^50^ and BEGDB^69^ web pages. All the benchmark interaction energies correspond to CCSD(T)/CBS calculations (with BSSE corrections). Table S4 specifies the complexes considered for this comparison and gives the values of the benchmark interaction energies as well as those calculated with the PM6, PM6-D3H4 and PM6-FGC approaches. The corresponding absolute errors are displayed in Figure 10, and the calculated MAEs are 10.6, 1.4, and 2.9 kJ/mol for PM6, PM6-D3H4, and PM6-FGC, respectively. Our method significantly improves the results of the PM6 Hamiltomian; but in general, it shows worse performance than the PM6-D3H4 method, although we notice that most of the complexes included in this comparison were used in the parameterization of the PM6-D3H4 method. Anyway, this comparison shows important information to assess the transferability of our corrections and provides guidelines for future improvements. We analyze these issues in the next paragraphs.

As shown in Table S4, systems 1–4 correspond to complexes of methylamine and *N*-methylacetamide (denoted in BEGDB as peptide). For these systems, our results are worse than the PM6-D3H4 results. Notice that the training set used for the parameterization of the PM6-D3H4 method was the S66x8 database,^25^ which includes systems 1–14. For system 6, the acetamide dimer, the performance of our method is clearly unsatisfactory. Interestingly, the PM6-FGC interaction energy calculated for the complex of pentane/acetamide (−14.8 kJ/mol), system 14 in Figure 10, is in excellent agreement with the S66 benchmark data (−14.7 kJ/mol). The interactions of pentane with *N*-methylacetamide and with acetic acid, systems 12 and 13, respectively, are also very well described by our method. These results are in line with previous work^47^ wherein pairwise analytical potentials obtained from fittings involving the CF_4_/NH_3_ and the CF_4_/HCOOH systems reproduced ab initio IPECs calculated for the CF_4_/HCONH_2_ complex.

The results discussed in the previous paragraph clarify an interesting feature regarding transferability in the studied systems. Although parameters obtained from NH_3_/CH_4_ fittings may be transferable to model −NH_2_/alkane interactions, other parameters obtained with NH_3_ may not be transferable to amines and amides. The description of noncovalent interactions involving the −NH_2_ group in amines and amides could be improved by including new representative compounds, namely, methylamine and acetamide. The water dimer is another challenging case; we did not succeed in developing corrections as defined in eqs 1 and 2. One way to overcome this problem involves the inclusion of pseudoatoms. These issues will be considered in future work.

It is important to notice that the interaction energies calculated at the B3LYP-D3/def2-TZVP level for methylamine and acetamide dimers (−18.0 and −70.1 kJ/mol, respectively) are in very good agreement with the reference CCSD(T)/CBS(haTZ) values, (−17.5 and −68.8 kJ/mol, respectively).^69^ For the sake of comparison, the corresponding CCSD(T)/aug-cc-pVTZ interaction energies are −16.7 and −65.9 kJ/mol, respectively. These results reinforce the use of B3LYP-D3/def2-TZVP data as the benchmark for our parameterizations.

For the hydrocarbon systems considered in Figure 10 (systems 7–11 and 16–23; see also Table S4), our method predicts reasonably good interaction energies. There are, however, some deviations for complexes involving neopentane (8 and 9). Finally, for system 15, which corresponds to the formaldehyde dimer, the interaction energy calculated with our method has an absolute error larger than that obtained with PM6. This indicates that the parameters of the carbonyl group developed from fittings on the formic acid dimer are not transferable to aldehydes and ketones, which supports the idea of using corrections for specific functional groups.

**Figure 11:**
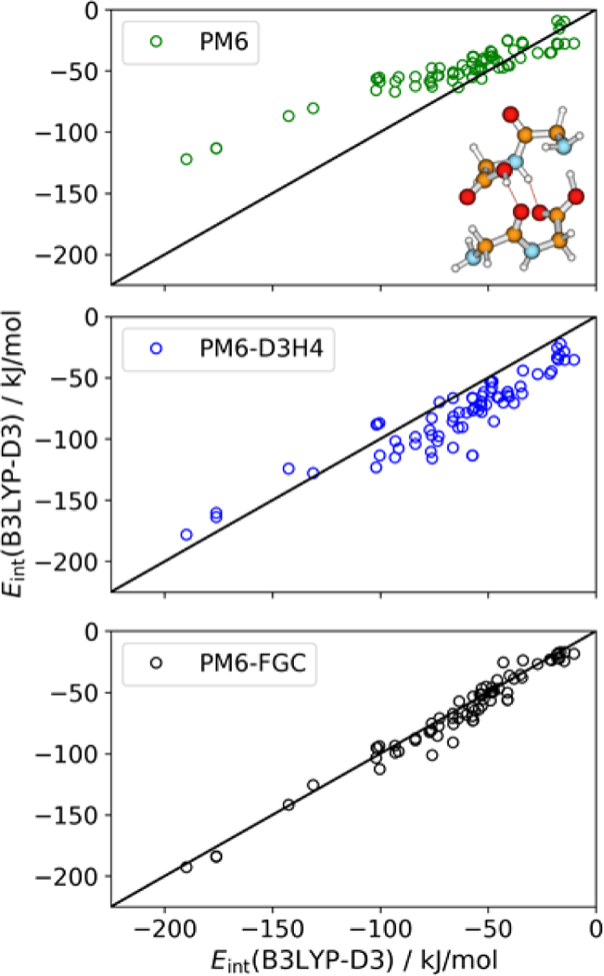
Linear correlations between the B3LYP-D3/def2-TZVP interaction energies and those calculated with PM6, PM6-D3H4, and PM6-FCG, for conformers of the diglycine dimer. The molecular drawing corresponds to the conformer with the largest interaction strength as calculated at the DFT level.

Even though the present corrections cannot accurately describe some interactions involving the −NH_2_ group, it is important to assess the performance of the method on larger systems. Because our purpose is to derive corrections for biological systems, here, we have studied complexes of diglycine and dialanine. Specifically, we have considered the dimers of these dipeptides as well as the trimer of diglycine. In this work, conformers of these complexes were found by automated exploration of their potential energy surfaces at the PM6-D3H4 level, using AutoMeKin,^63−66^ which has an interface with the MOPAC2016 program.^24^ These searches involved changes in both intramolecular and intermolecular conformations and led to 77 and 90 different conformers for diglycine and dialanine dimers, respectively, and 146 conformers for the diglycine trimer. Figure 11 shows linear correlations between the interaction energies calculated at the B3LYP-D3/def2-TZVP level and those evaluated with the SQM methods considered here, for the complexes of the diglycine dimer. The diagonal straight lines represent the case of perfect correlation. As can be seen, our corrections significantly improve the performance of the PM6 Hamiltonian (shown as green open circles in Figure 11). As shown in Table 2, the mean absolute errors (MAE) and mean bias errors (MBE) calculated for both PM6-FGC parameter sets are substantially smaller than those computed for PM6. The bias error is calculated as the mean of the differences between the reference values and the SQM values. In the case of the diglycine dimer, the MAE calculated for PM6-D3H4 is very similar to that of PM6 (∼18 kJ/mol). Likewise, the bias values for these two methods are very similar to each other in absolute value. However, the PM6 method underestimates the strength of the interactions (negative MBE), especially for the most attractive conformers of the complexes, whereas the PM6-D3H4 method exhibits overestimation (positive MBE), especially for conformers showing from low to moderate interaction strengths.

**Table 2:** Statistical Parameters^a^ of the Linear Correlations between B3LYP-D3/def2-TZVP Interaction Energies and the Different PM6 SQM Interaction Energies Calculated for the Conformers of the Diglycine and Dialanine Dimers, as Well as the Diglycine Trimer

	diglycine dimer		dialanine dimer		diglycine trimer	
	MAE	MBE	MAE	MBE	MAE	MBE
PM6	17.9	–15.0	14.3	–14.0	10.8	–5.1
PM6-D3H4	18.6	15.5	20.3	19.5	25.3	25.3
PM6-FGC	6.0	3.2	8.7	8.7	9.1	8.7

a MAE and MBE values are given in kJ/mol.

**Figure 12:**
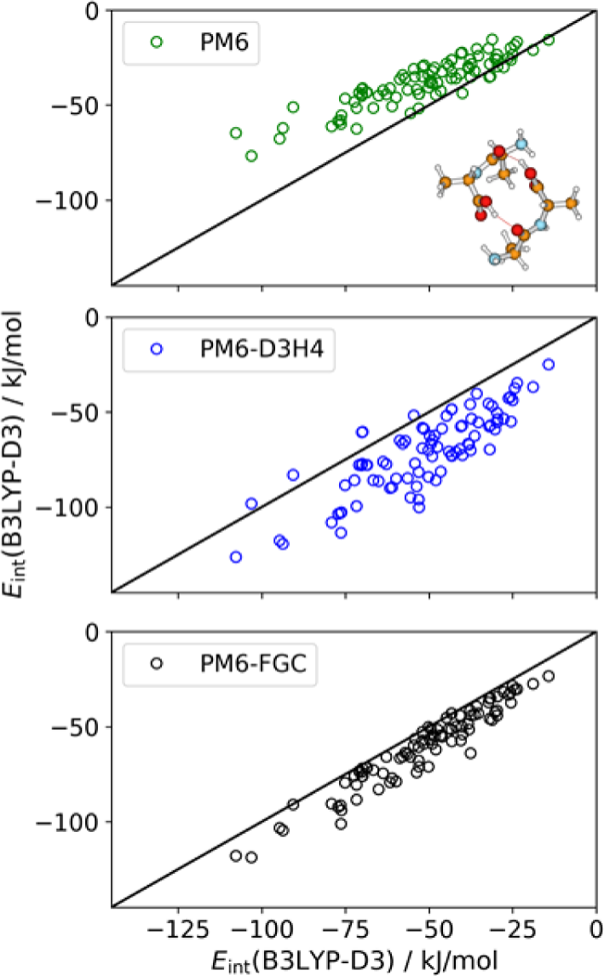
Linear correlations between the B3LYP-D3/def2-TZVP interaction energies and those calculated with PM6, PM6-D3H4, and PM6-FCG, for conformers of the dialanine dimer. The molecular drawing corresponds to the conformer having the largest interaction strength as calculated at the DFT level.

**Figure 13:**
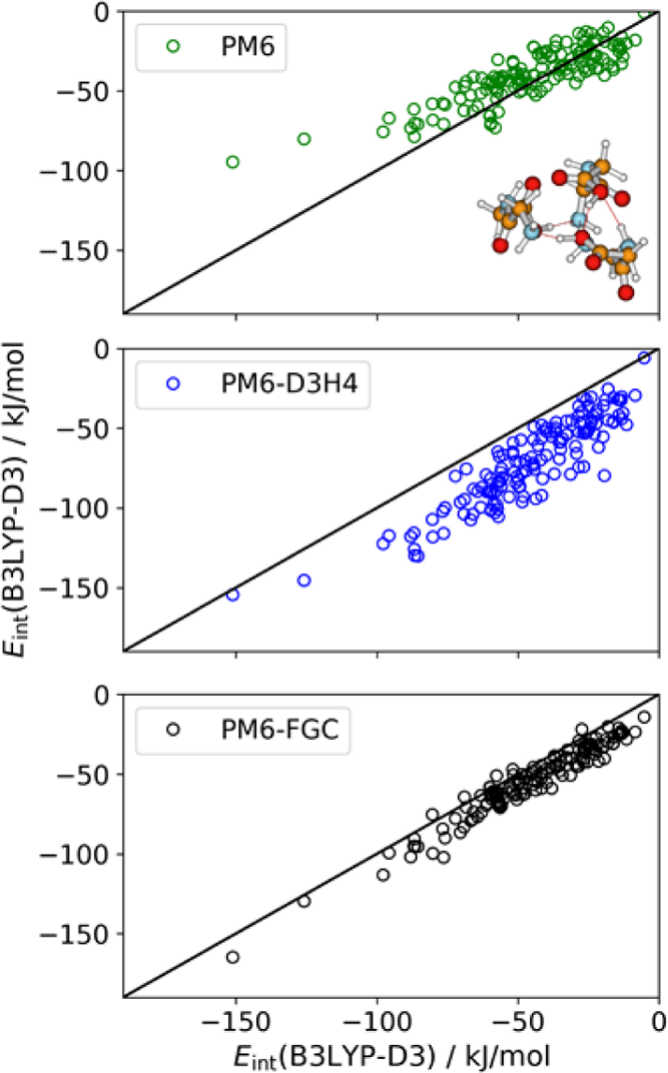
Linear correlations between the B3LYP-D3/def2-TZVP interaction energies and those calculated with PM6, PM6-D3H4, and PM6-FCG, for conformers of the diglycine trimer. The molecular drawing corresponds to the conformer having the largest interaction strength as calculated at the DFT level.

The linear correlations evaluated for the conformers of the dialanine dimer are depicted in Figure 12. The MAE and MBE values (Table 2) calculated for PM6-D3H4 are somewhat larger than those computed for PM6. Again, the PM6 and the PM6-D3H4 methods lead to underestimation and overestimation, respectively, of the interaction energies. Finally, Figure 13 depicts the linear correlations for the diglycine trimer. For this system, the calculated interaction energies correspond to the difference between the energy of the trimer and that of separated dimer and monomer. As can be seen, the figure shows the same trend observed in Figures 11 and 12. The PM6 method underestimates the strength of the interaction energies whereas PM6-FGC and, especially, PM6-D3H4 overestimate it. The MAE calculated for PM6-D3H4 is more than twice the PM6 or PM6-FGC MAE. However, the errors in the PM6 interaction energies for the most attractive complexes are remarkable. Although a direct comparison with PM6-D3H4 results should be taken with caution because the latter corrections were determined from a CCSD(T)/CBS reference; the results of the present work provide evidence of deficiencies in the PM6-D3H4 method (and other SQM methods). These deficiencies are mainly the result of using, for the parameterization scheme, a data set (i.e., the S66 database^25,27^) that does not include sufficient orientations of the interacting molecules.

Finally, we would like to point out that our corrections may be problematic in the study of chemical reactions, since, in general, SQM methods do not predict accurate reaction barriers. To overcome this problem, we recommend the use of specific reaction parameters (SRPs), following the strategy pioneered by Truhlar and co-workers.^70^ SRPs should be developed and applied to the atoms involved in the reaction, and our corrections should not be used for these atoms.

## 4 Conclusions

We have presented a new strategy, that is, the PM6-Functional Group Corrections (PM6-FGC) method, to develop analytical corrections for semiempirical quantum mechanical methods, aimed at improving the description of noncovalent interactions. For this proof-of-concept presentation, we have selected the PM6 SQM method. Employing this Hamiltonian and the B3LYP-D3/def2-TZVP level for benchmarking, we calculated intermolecular potential energy curves for several orientations of pairs of small molecules, which are selected as representatives of different functional groups. Specifically, we have considered ammonia, formic acid, and methane, which result in a total of six molecular pairings. A simple mathematical expression, which should be considered as a practical way, without any physical meaning, is used to represent the corrections to improve the performance of SQM methods. The parameters of the analytical corrections were evaluated from fits to differences between interaction energies calculated at the reference level and those evaluated at the SQM level.

Intermolecular potential energy curves (IPECs) were also evaluated at the CCSD(T)/aug-cc-pVTZ, including counterpoise correction for BSSE. The agreement between the B3LYP-D3 and the corresponding CCSD(T) curves was excellent. This result together with the fact that we will need to perform extensive benchmark calculations to extend our method to other functional groups, in order to develop a universally applicable method for peptides and other biological systems, led us to select the DFT method as the benchmark level for the parameterizations.

In general, the IPECs obtained with the method parameterized in this work, namely, PM6-FGC, are in good agreement with those determined at the DFT level, and significantly improve those provided by successfully corrected SQM methods. In this way, this work emphasizes the importance of including, in the databases, sufficient orientations of the interacting molecules; this fact is crucial to develop well-balanced corrections.

Even though the corrections proposed in this work are based on general pairwise functions that include a significant number of parameters, several validation tests suggest that our parameterization strategy is not affected by overfitting, at least at a significant level. A comparison of interaction energies calculated for a series of complexes of well-known data sets has revealed important warnings concerning parameter transferability. In particular, we found that the ammonia dimer is not a good representative for the parameterization of corrections for some interactions involving the −NH_2_ group in amines and amides. This issue will be faced in future work. However, using the present set of parameters, we evaluated interaction energies for a large number of conformers of diglycine and dialanine dimers, and diglycine trimer, obtaining very good results in comparison with those predicted by PM6 and PM6-D3H4.

Although the method described here involves simple pairwise functions, more accurate corrections could be developed by using or adding alternative functions, as well as by introducing three-body terms of the type of the Axilrod–Teller–Muto potential.^23,40^ We are continuing with our efforts to improve and extend our corrections to other functional groups, relevant to biological compounds, and we intend to implement them in the MOPAC program.^24^ Meanwhile, a Python script to compute PM6-FGC corrections is available upon request.

## Supporting Information Available

Parameters obtained from fittings to B3LYP-D3/def2-TZVP–PM6 interaction energy differences; parameters obtained for the formic acid dimer using 4 atom types and 3 atom types; interaction energies for several complexes of well-known data sets; comparisons of IPECs calculated at the CCSD(T)/aug-cc-pVTZ and B3LYP-D3/def2-TZVP; comparison of IPECs for orientations not shown in the article ; comparison of IPECs for the formic acid dimer obtained with corrections determined using 5, 4, and 3 atom types; and comparison of IPECs for 10 random orientations of the formic acid dimer (PDF)
