# Supplementary material for: New Approach for Correcting Noncovalent Interactions in Semiempirical Quantum Mechanical Methods: The Importance of Multiple-Orientation Sampling
Source: J Chem Theory Comput. 2021 Aug 23;17(9):5556–67. doi: 10.1021/acs.jctc.1c00365 (PMC8486165; doi:10.1021/acs.jctc.1c00365)
Supplement: Supplementary file 1 — ct1c00365_si_001.pdf [file ct1c00365_si_001.pdf]

Supporting Information for

A New Approach for Correcting Noncovalent Interactions in Semiempirical Quantum Mechanical Methods. The Importance of Multiple-Orientation Sampling

*Sergio Pérez-Tabero, Berta Fernández, Enrique M. Cabaleiro-Lago, Emilio Martínez-Núñez and Saulo A. Vázquez**

Departamento de Química Física, Facultade de Química, Universidade de Santiago de Compostela, 15782 Santiago de Compostela, Spain.

In this document, tables are presented before figures.

Table S1. Parameters[a] obtained in this study from fits to B3LYP-D3 – PM6 interaction energy differences.

Atom pair	***A***	***B***	***C***	***n***	***d***
C–C	87222.6781703499	4.5192960449	–2614.2390222503	6	1.8
C–O	18423.1251143120	3.2165256155	–4.7565336414	10	1.7
C–OH	101988.8220580146	3.7620617158	–801.8979286559	6	1.7
C–HO	–118682.0300621045	5.3124974616	49.9211435603	4	1.2
C–HCO	–91515.9156668177	5.3134822945	5.6811250600	2	1.2
O–O	157533.3119267652	3.8363490696	–249.6964065606	9	1.7
O–OH	229576.1122743190	3.7398102896	–1494.5123518379	6	1.7
O–HO	–12321.2844572512	5.1464744095	–106.3638810776	3	1.0
O–HCO	7911.4730614547	3.4463958590	–735.0028283044	9	1.2
OH–OH	282168.5215761239	3.7405298249	–1073.4903717942	5	1.7
OH–HO	–3746.8891857739	3.4364552152	–49.8028148968	4	1.0
OH–HCO	3900.4353604300	2.9066422385	–380.8650480007	8	1.2
HO–HO	2029.7914153477	3.0685212894	27.0433227950	2	1.0
HO–HCO	10653.6465827195	4.2574799189	–396.7704915296	11	1.0
HCO–HCO	9870.5095997493	3.4861656955	–289.7235420067	5	1.0
N–N	14325.7433949299	2.3474982183	0.0	0	1.8
HN–N	67180.6938579479	4.0291906191	–2542.4108143032	6	1.2
HN–HN	2669.0457913742	2.6179842324	–301.4938231690	6	1.2
CT–CT	15857.1701602370	2.6980129877	–770.4222566934	5	1.8
CT–HC	1146.3278506151	2.2146125958	–1170.1797569593	6	1.2
HC–HC	18261.8993616442	4.3048851482	–522.7261176189	10	1.2
C–N	8824.6374645503	2.7387802474	40.0451859169	4	1.8
C–HN	–9896.4961081155	4.3777416739	–283.1284987128	5	1.2
O–N	189991.8017198780	3.6348674855	–0.0066170660	8	1.8
O–HN	11946.3431019849	4.2125802231	–782.9420790391	8	1.2
OH–N	10971.8258111343	2.3045860697	–1499.8948318794	6	1.8
OH–HN	30103.3944495938	5.0405312142	–313.4604773768	6	1.2
HO–N	294065.3724309836	4.5821919300	–4264.6523403123	6	1.2
HO–HN	1989.0390506399	2.7000114780	–114.0461092908	8	1.2
HCO–N	8856.4806583822	3.7125803660	–527.1736982525	10	1.2
HCO–HN	4914.7612479035	3.5584587531	–108.0011048550	6	1.2

[a] The units are such that the potential energy is in kJ/mol and distances in Å.

Table S2. Parameters obtained for the formic acid dimer using 4 atom types. [a]

Atom pair	*A*	*B*	*C*	*n*	*d*
C–C	22408.8544303881	3.4465345418	–950.7939379409	4	1.8
C–O	69277.5542364235	4.1702334762	2094.1389964922	7	1.7
C–HO	–42553.1826225501	4.0857645865	557.3220188713	5	1.2
C–HCO	–59910.7120763586	4.4892910651	799.9193671203	7	1.2
O–O	64592.1369713109	3.2999068357	–183.6670261481	4	1.7
O–HO	24670.3889752773	4.1295196868	–644.0828580278	5	1.0
O–HCO	–19997.2931961984	4.6610258492	362.8629755965	5	1.2
HO–HO	1606.1495593326	2.8489639131	30.0917630272	2	1.0
HCO–HCO	17226.6706394802	4.7657674333	–330.6576549135	12	1.0

[a] The units are such that the potential energy is in kJ/mol and distances in Å.

Table S3. Parameters obtained for the formic acid dimer using 3 atom types. [a]

Atom pair	*A*	*B*	*C*	*n*	*d*
C–C	–9116.8005278657	2.0792963700	3394.2455735437	5	1.8
C–O	649.0175304136	2.1775769979	1349.2249963131	4	1.7
C–H	31336.4312036947	3.1946098034	–2331.5728643242	5	1.2
O–O	653671.5687567827	4.0063379104	–4190.0825545768	5	1.7
O–H	8618.8758225370	3.5540240457	–760.8050497928	7	1.0
H–H	2429.3645285537	5.4997006357	143.2754503083	4	1.0

[a] The units are such that the potential energy is in kJ/mol and distances in Å.

Table S4. Interaction energies (in kJ/mol) for several complexes of well-known data sets.[a]

Number	System	Benchmark	PM6	PM6-D3H4	PM6-FGC
1	MeNH2/MeNH2[b]	-17.5	-7.7	-19.0	-13.1
2	MeNH2/peptide[b]	-22.8	-16.1	-25.7	-18.8
3	Peptide/MeNH2[b]	-31.5	-17.5	-31.4	-27.6
4	Peptide/peptide[b]	-36.3	-24.7	-36.8	-30.1
5	AcOH/AcOH[b]	-80.8	-46.4	-76.8	-79.4
6	AcNH2/AcNH2[b]	-68.7	-51.8	-70.7	-49.6
7	Pentane/pentane[b]	-15.7	-2.7	-14.5	-16.1
8	Neopentane/pentane[b]	-10.8	-2.9	-10.8	-13.6
9	Neopentane/neopentane[b]	-7.4	-2.3	-8.7	-11.2
10	Cyclopentane/neopentane[b]	-10.0	-2.8	-10.5	-13.3
11	Cyclopentane/cyclopentane[b]	-12.5	-1.6	-9.8	-13.4
12	Peptide/pentane[b]	-17.7	-5.3	-17.2	-17.6
13	Pentane/AcOH[b]	-12.1	-5.5	-15.0	-12.3
14	Pentane/AcNH2[b]	-14.7	-6.4	-16.5	-14.8
15	Formaldehyde dimer[c]	-19.0	-15.9	-18.2	-10.7
16	Methane/ethane[c]	-3.5	-0.6	-4.2	-2.6
17	Methane/ethane[c]	-2.6	-0.5	-3.5	-2.1
18	Ethane dimer[d]	-5.6	-1.3	-5.9	-4.8
29	Propane dimer[d]	-8.3	-1.3	-7.4	-7.5
20	Butane dimer[d]	-12.1	-2.0	-11.7	-12.0
21	Pentane dimer[d]	-15.8	-2.7	-15.1	-16.6
22	Hexane dimer[d]	-19.2	-2.9	-18.2	-20.5
23	Heptane dimer[d]	-23.2	-3.6	-20.0	-24.6

[a] Benchmark interaction energies correspond to CCSD(T)/CBS calculations as detailed in the references below.

[b] Benchmark data from the BEGDB web page[1] and Řezáč et al.[2]

[c] From the BEGDB web page[1] and Řezáč et al.[3]

[d] From the GMTKN55 web page.[4]

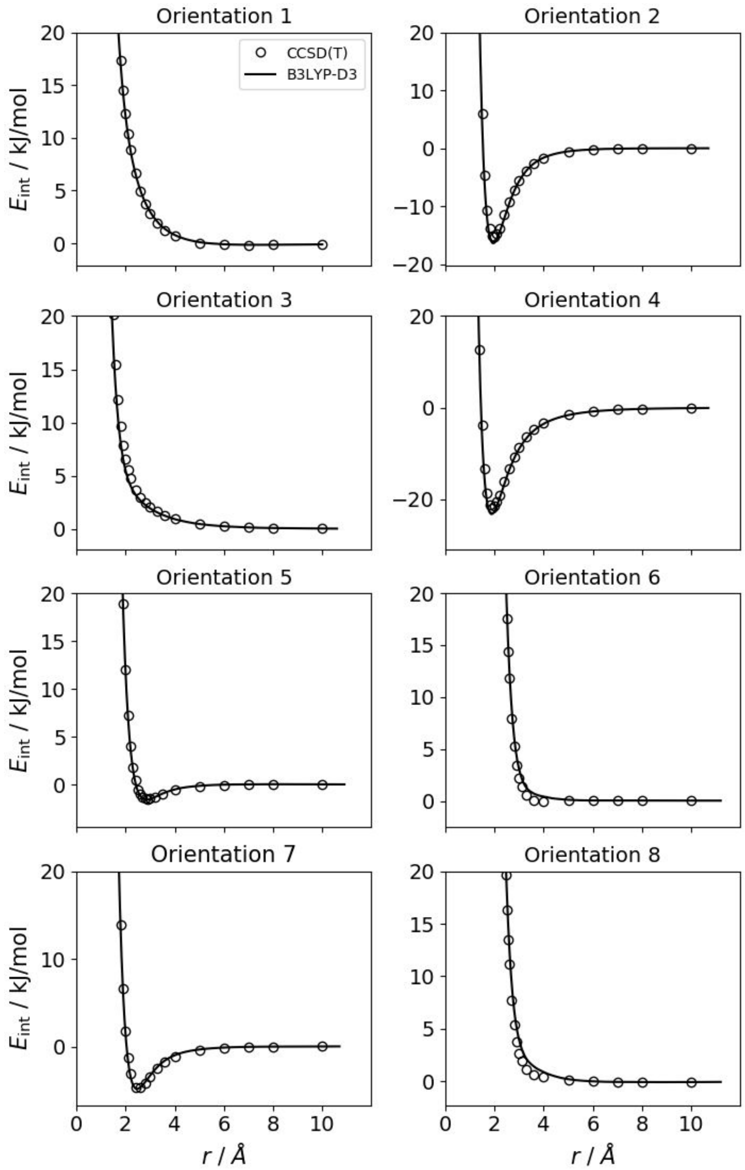

Figure S1. Comparison of CCSD(T) and DFT IPECs for orientations 1-8 of the formic acid dimer.

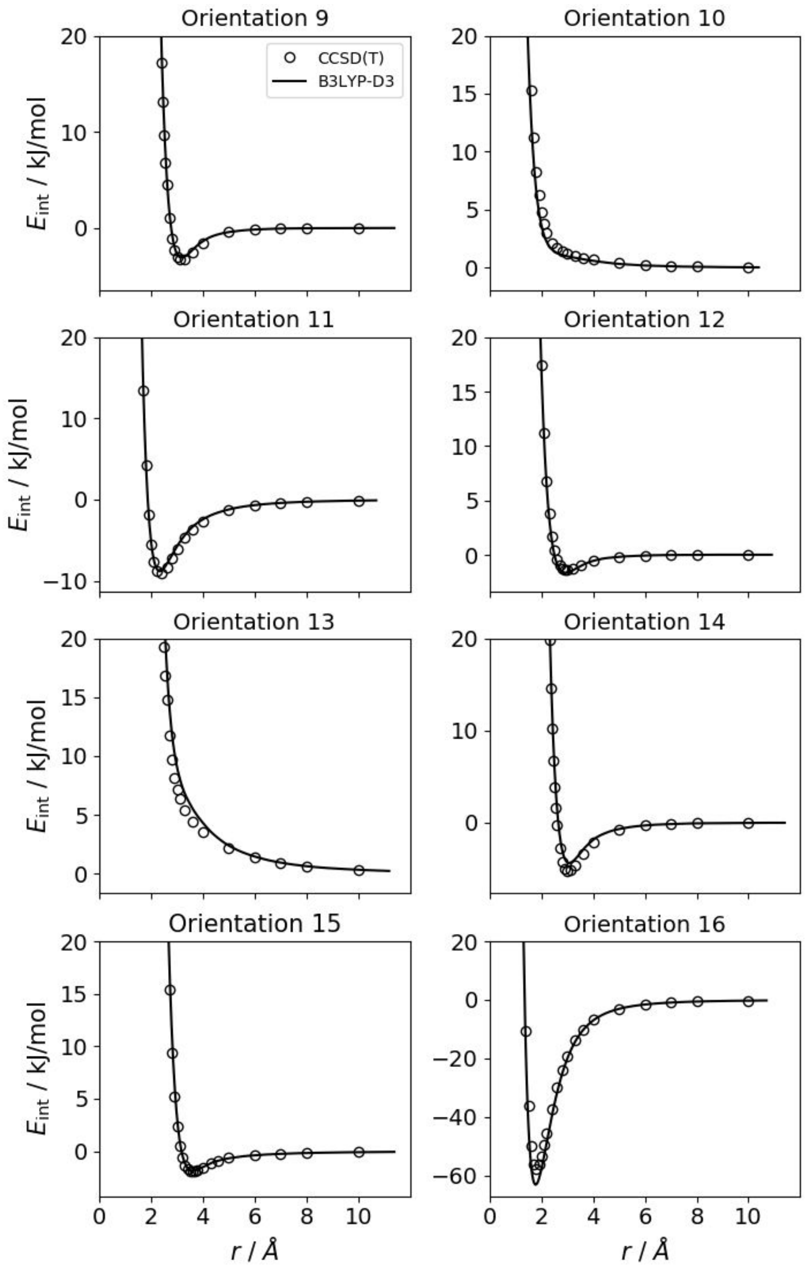

Figure S2. Comparison of CCSD(T) and B3LYP-D3 IPECs for orientations 9 - 16 of the formic acid dimer.

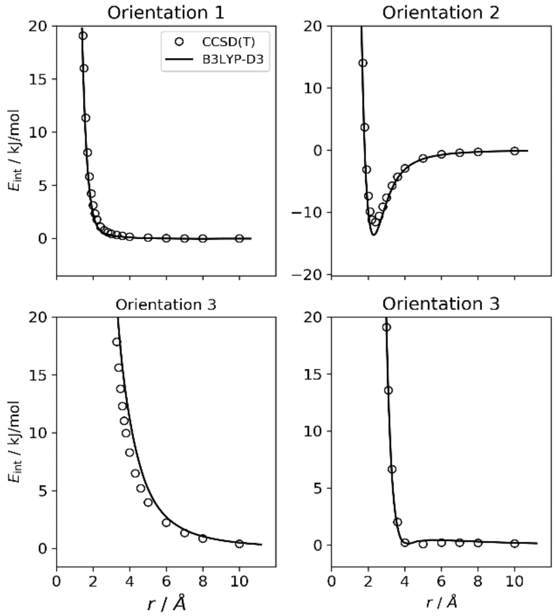

Figure S3. Comparison of CCSD(T) and B3LYP-D3 IPECs for the orientations of the ammonia dimer.

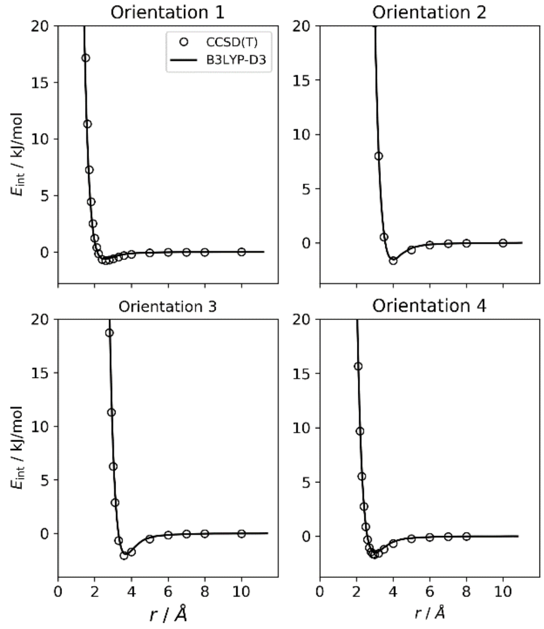

Figure S4. Comparison of CCSD(T) and B3LYP-D3 IPECs for the orientations of the methane dimer.

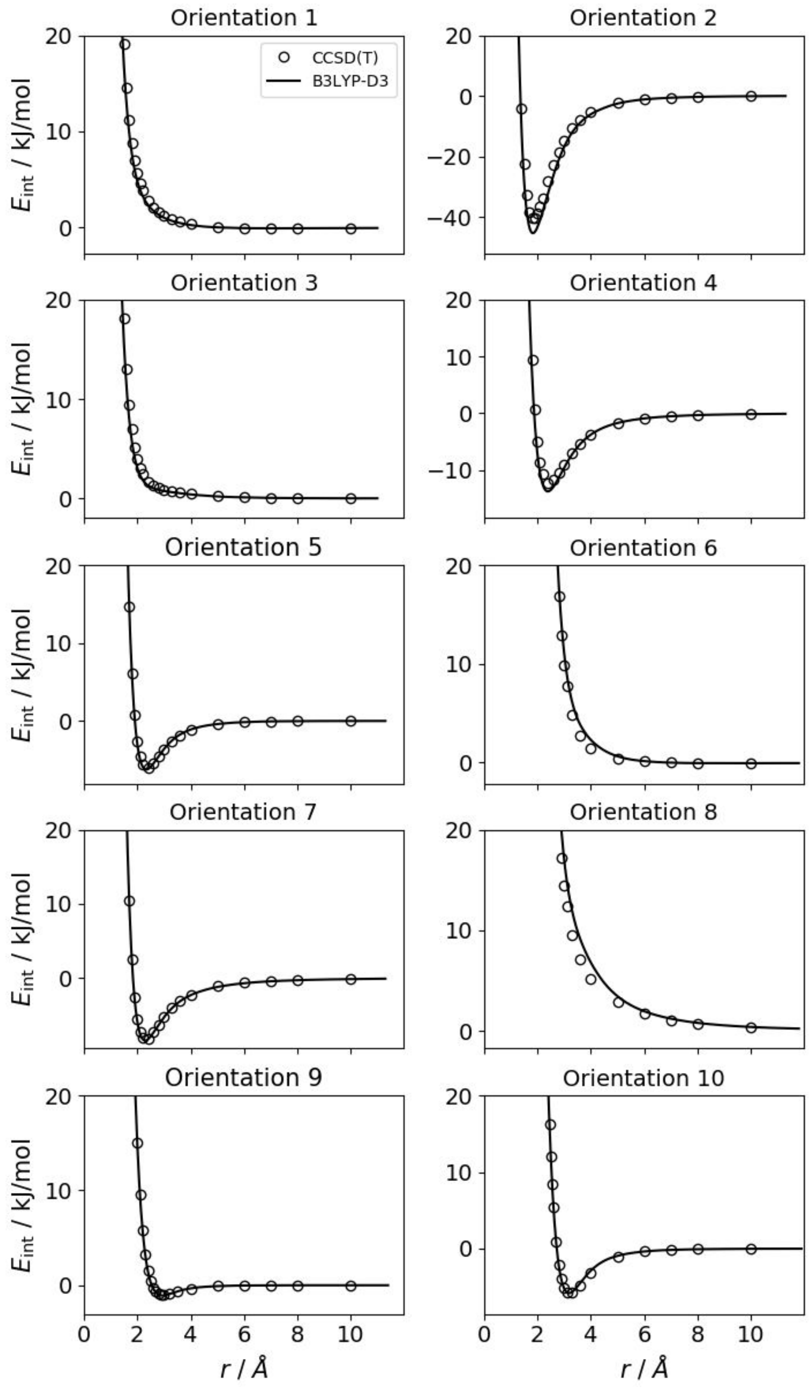

Figure S5. Comparison of CCSD(T) and B3LYP-D3 IPECs for the orientations of the $HCOOH/NH_3$ complex.

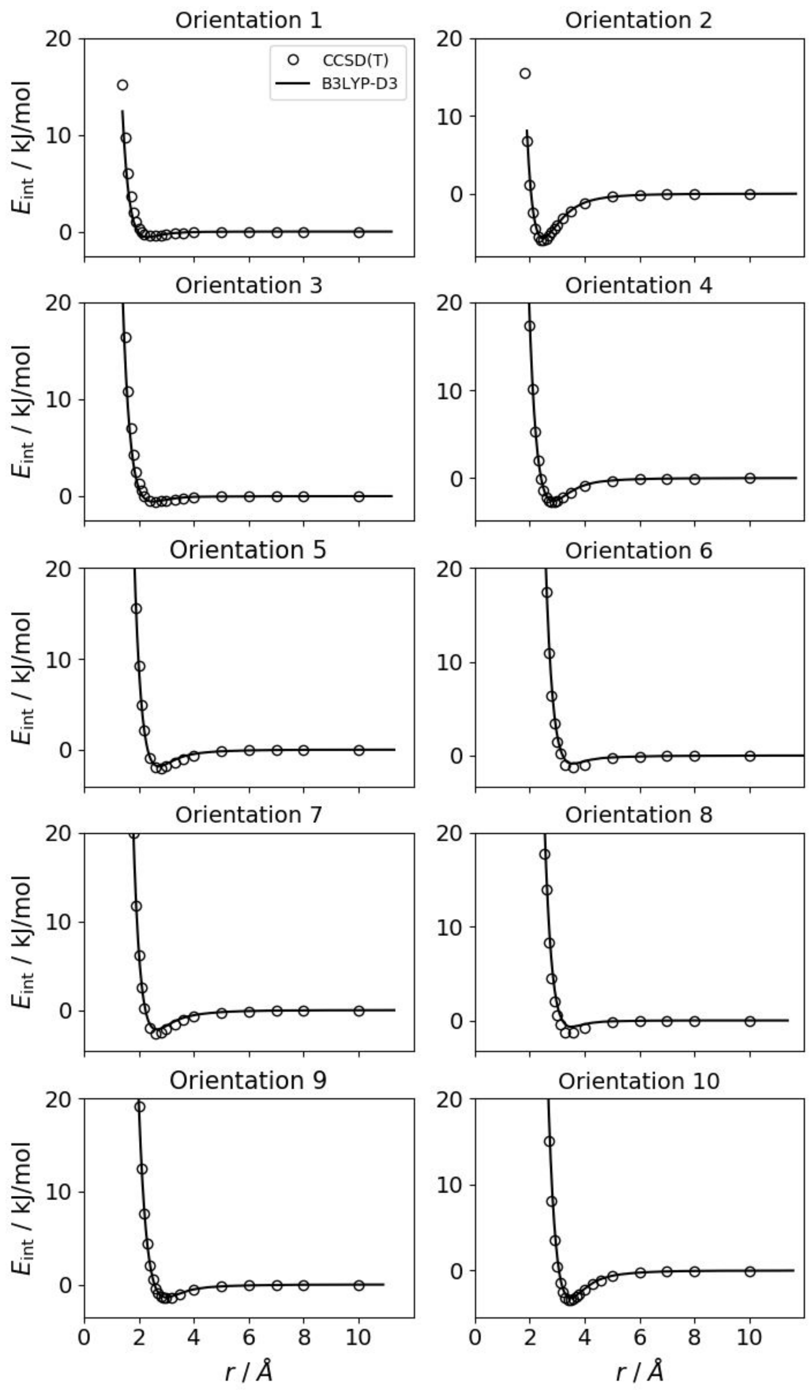

Figure S6. Comparison of CCSD(T) and B3LYP-D3 IPECs for the orientations of the $HCOOH/CH_4$ complex.

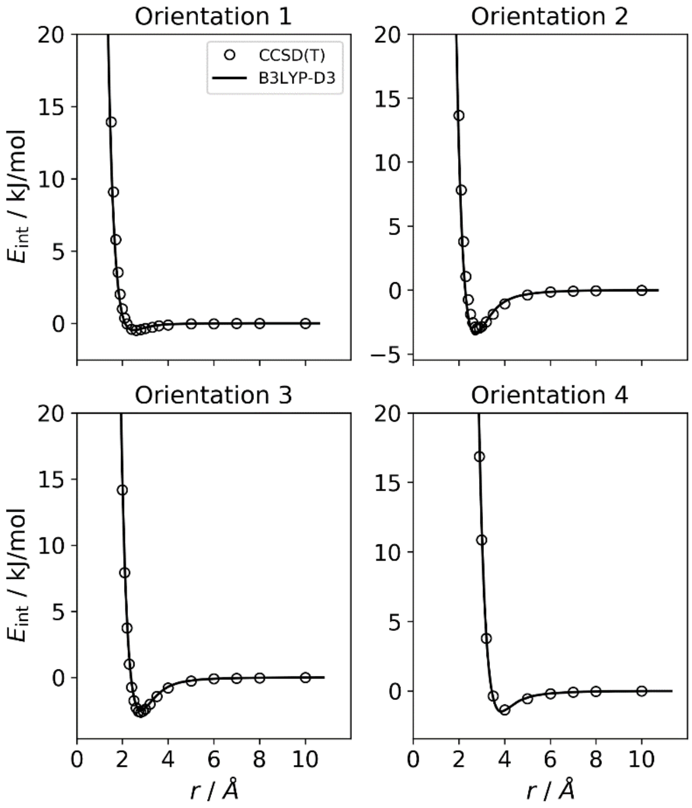

Figure S7. Comparison of CCSD(T) and B3LYP-D3 IPECs for the orientations of the NH_3/CH_4 complex.

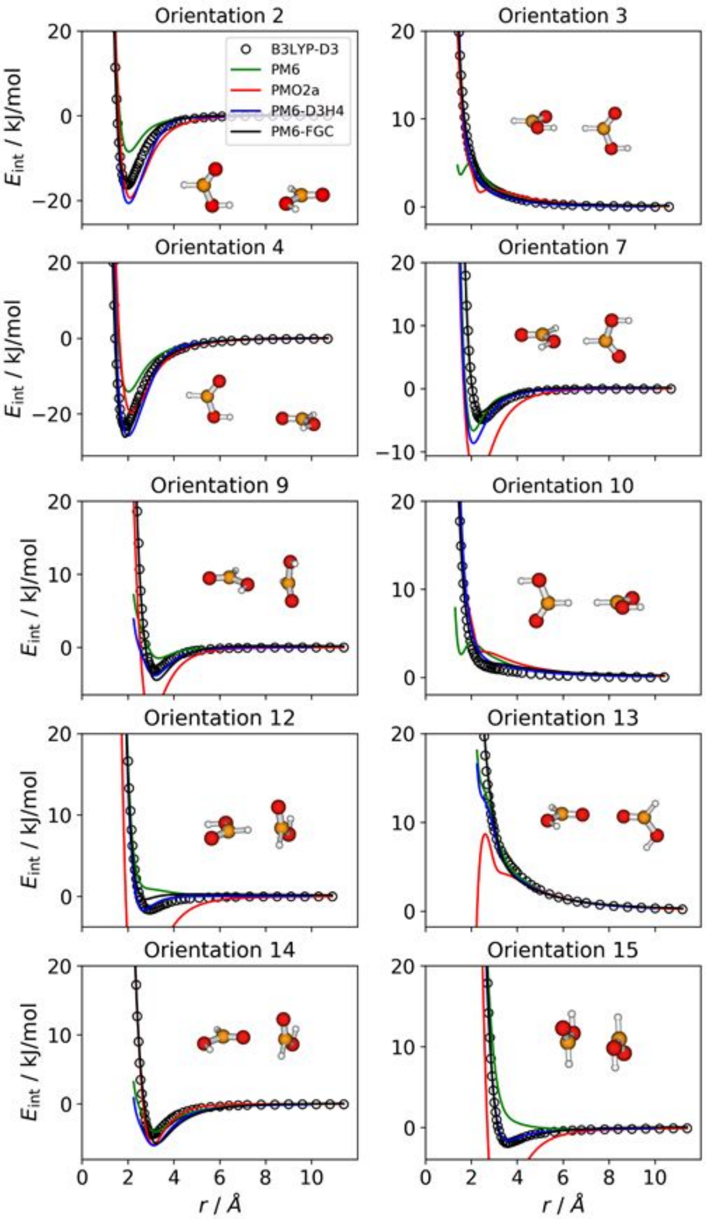

Figure S8. Comparison of IPECs for the orientations of the formic acid dimer not shown in the manuscript.

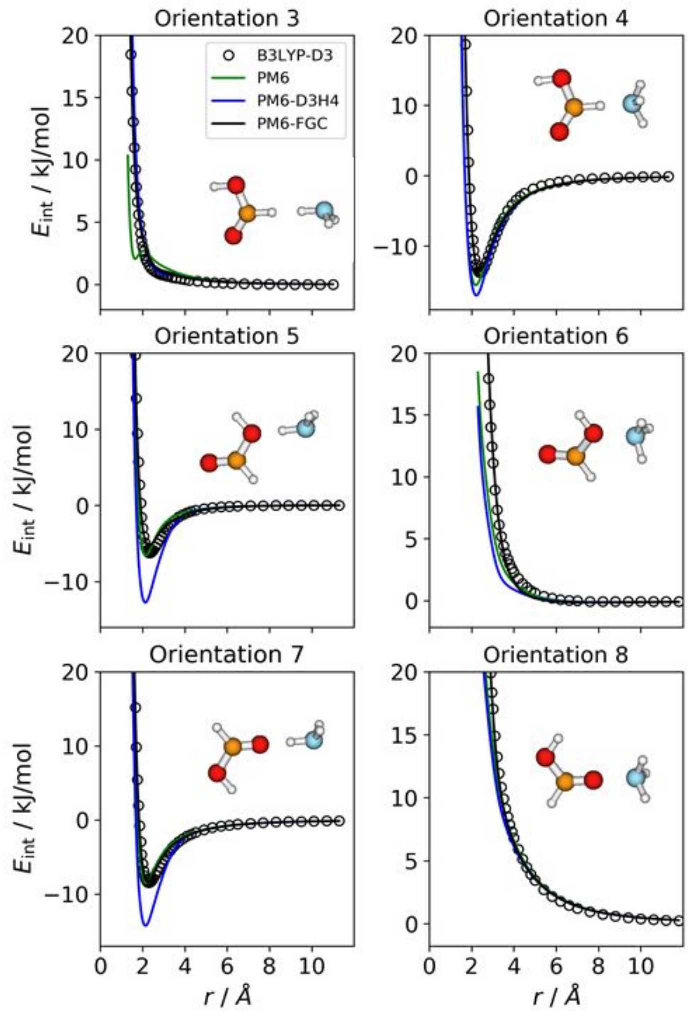

Figure S9. Comparison of IPECs for the orientations of the HCOOH/NH_3 complex not shown in the manuscript.

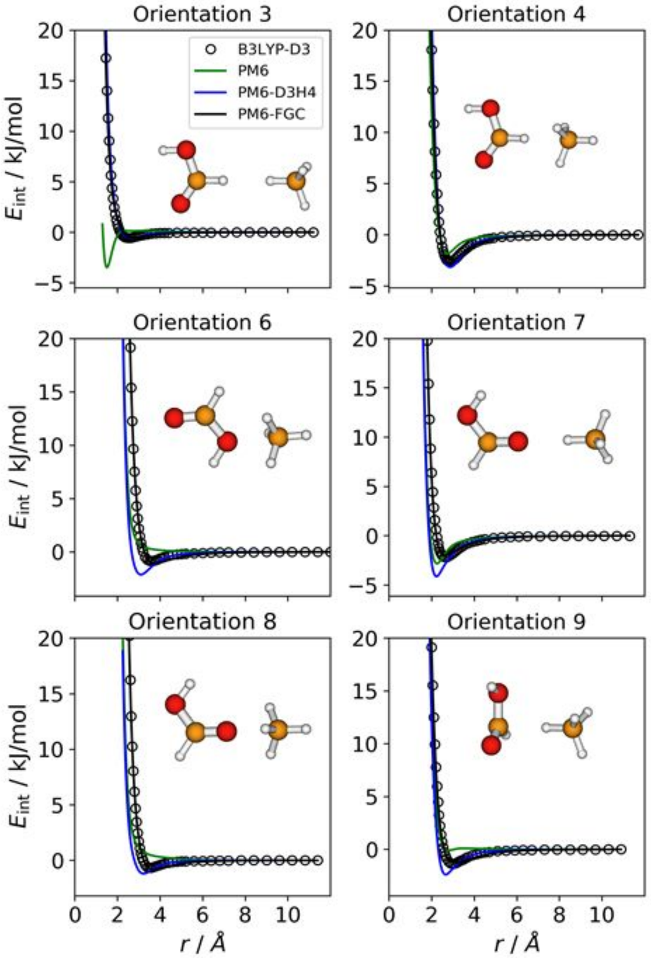

Figure S10. Comparison of IPECs for the orientations of the $HCOOH/CH_4$ complex not shown in the manuscript.

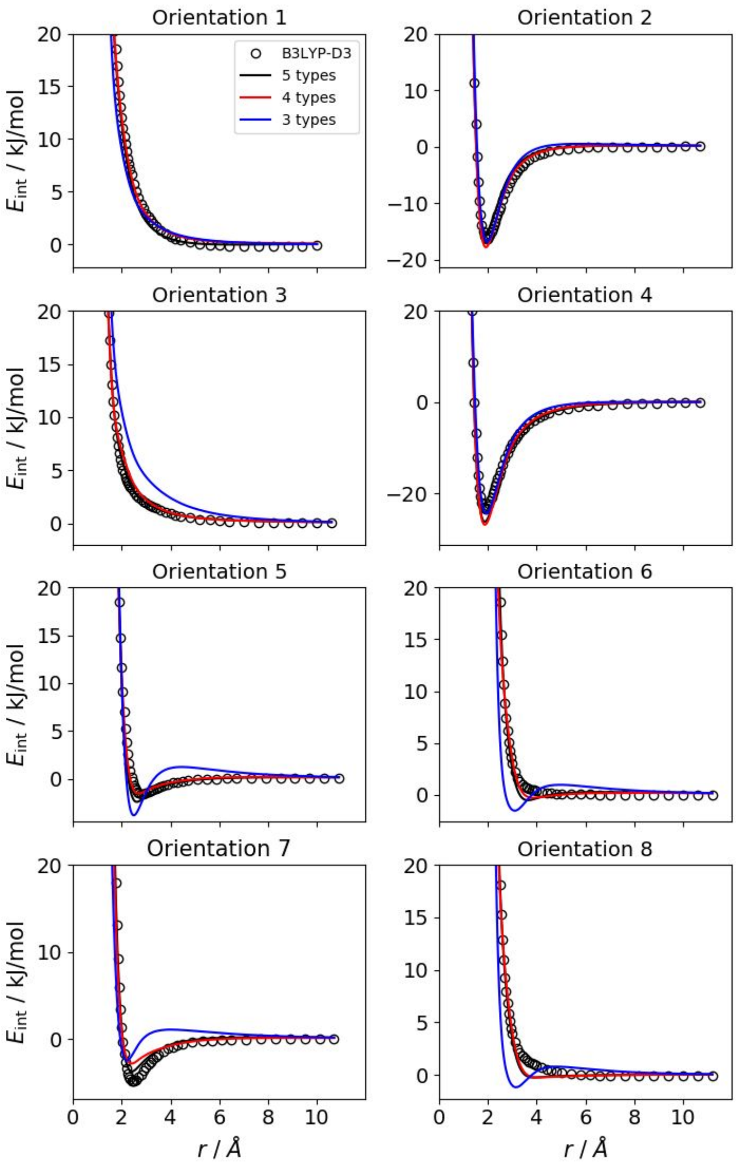

Figure S11. Comparison of IPECs for orientations 1-8 of the formic acid dimer, obtained with corrections determined using 5 atom types (black curves), 4 atom types (red curves), and 3 atom types (blue curves). The reference IPECs are displayed as open circles.

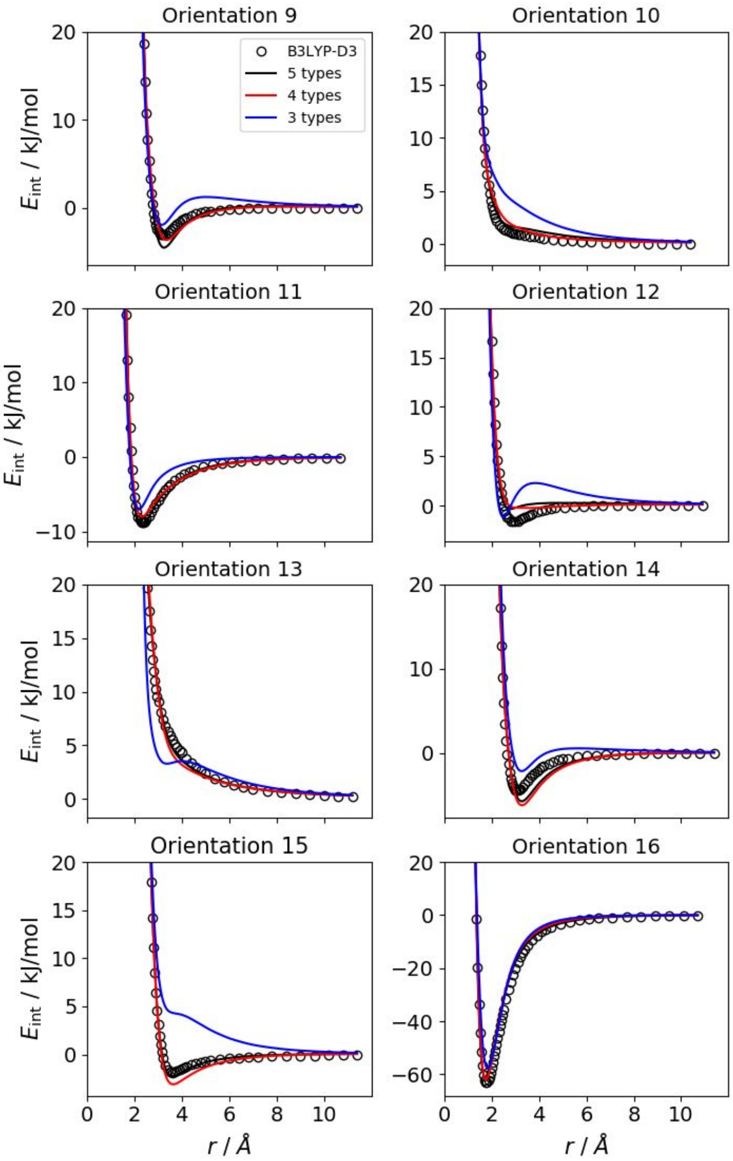

Figure S12. Comparison of IPECs for orientations 9-16 of the formic acid dimer, obtained with corrections determined using 5 atom types (black curves), 4 atom types (red curves), and 3 atom types (blue curves). The reference IPECs are displayed as open circles.

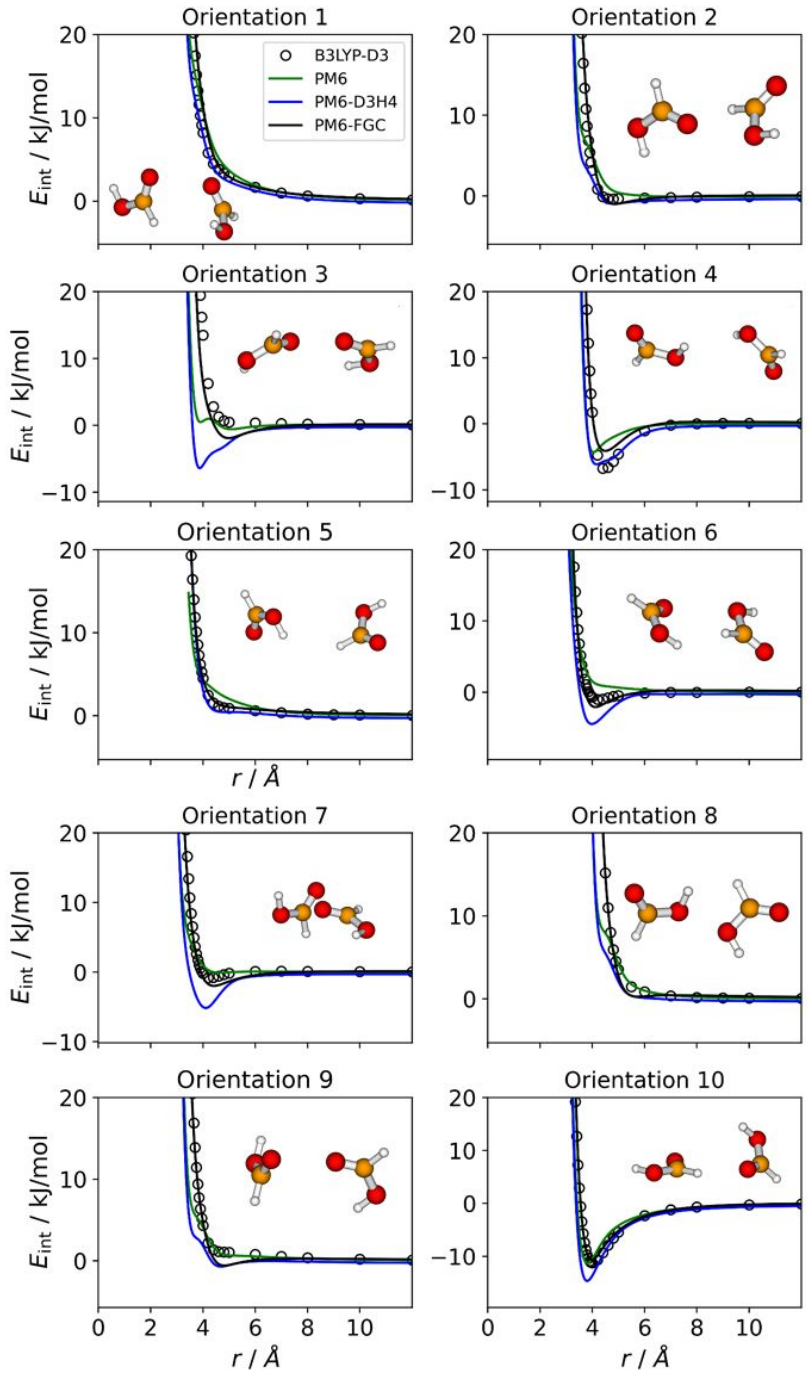

Figure S13. Comparison of IPECs for 10 random orientations of the formic acid dimer. These orientations were not included in the fittings. Interaction energies are plotted here as a function of the distance r between the center of masses of the monomers.

REFERENCES

1. Řezáč, J.; Jurečkab, P.; Riley, K. E.; Černý, J.; Valdes, H.; Pluháčková, K.; Berka, K.; Řezáč, T.; Pitoňák, M.; Vondrášek, J.; Hobza, P., Quantum Chemical Benchmark Energy and Geometry Database for Molecular Clusters and Complex Molecular Systems (www.begdb.com): A Users Manual and Examples. *Collect. Czech. Chem. Commun.* **2008,** *73*, 1261-1270.
2. Řezáč, J.; Riley, K. E.; Hobza, P., S66: A Well-balanced Database of Benchmark Interaction Energies Relevant to Biomolecular Structures. *J. Chem. Theory Comput.* **2011,** *7* (8), 2427-2438.
3. Řezáč, J.; Hobza, P., Describing Noncovalent Interactions beyond the Common Approximations: How Accurate Is the "Gold Standard," CCSD(T) at the Complete Basis Set Limit? *J. Chem. Theory Comput.* **2013,** *9* (5), 2151-2155.
4. Goerigk, L.; Hansen, A.; Bauer, C.; Ehrlich, S.; Najibi, A.; Grimme, S., A look at the density functional theory zoo with the advanced GMTKN55 database for general main group thermochemistry, kinetics and noncovalent interactions. *Phys. Chem. Chem. Phys.* **2017,** *19* (48), 32184-32215.
